# Circulation and characterization of seasonal influenza viruses in Cambodia, 2012‐2015

**DOI:** 10.1111/irv.12647

**Published:** 2019-06-28

**Authors:** Paul F. Horwood, Erik A. Karlsson, Srey Viseth Horm, Sovann Ly, Seng Heng, Savuth Chin, Chau Darapheak, David Saunders, Lon Chanthap, Sareth Rith, Phalla Y, Kim Lay Chea, Borann Sar, Amy Parry, Vanra Ieng, Reiko Tsuyouka, Yi‐Mo Deng, Aeron C. Hurt, Ian G. Barr, Naomi Komadina, Philippe Buchy, Philippe Dussart

**Affiliations:** ^1^ Virology Unit, Institute Pasteur in Cambodia Institute Pasteur International Network Phnom Penh Cambodia; ^2^ Australian Institute of Tropical Health and Medicine James Cook University Cairns Queensland Australia; ^3^ Communicable Disease Control Department Ministry of Health Phnom Penh Cambodia; ^4^ National Institute of Public Health Phnom Penh Cambodia; ^5^ Armed Forces Research Institute of Medical Sciences Bangkok Thailand; ^6^ Centers for Disease Control and Prevention Phnom Penh Cambodia; ^7^ World Health Organization Phnom Penh Cambodia; ^8^ WHO Collaborating Centre for Reference and Research on Influenza VIDRL, Peter Doherty Institute Melbourne Victoria Australia; ^9^ Monash University Melbourne Victoria Australia; ^10^ GlaxoSmithKline Vaccines R&D Intercontinental Singapore Singapore

**Keywords:** A(H1N1)pdm09, A(H3N2), Cambodia, influenza A virus, influenza B virus, surveillance

## Abstract

**Background:**

Influenza virus circulation is monitored through the Cambodian influenza‐like illness (ILI) sentinel surveillance system and isolates are characterized by the National Influenza Centre (NIC). Seasonal influenza circulation has previously been characterized by year‐round activity and a peak during the rainy season (June‐November).

**Objectives:**

We documented the circulation of seasonal influenza in Cambodia for 2012‐2015 and investigated genetic, antigenic, and antiviral resistance characteristics of influenza isolates.

**Patients/Methods:**

Respiratory samples were collected from patients presenting with influenza‐like illness (ILI) at 11 hospitals throughout Cambodia. First‐line screening was conducted by the National Institute of Public Health and the Armed Forces Research Institute of Medical Sciences. Confirmation of testing and genetic, antigenic and antiviral resistance characterization was conducted by Institute Pasteur in Cambodia, the NIC. Additional virus characterization was conducted by the WHO Collaborating Centre for Reference and Research on Influenza (Melbourne, Australia).

**Results:**

Between 2012 and 2015, 1,238 influenza‐positive samples were submitted to the NIC. Influenza A(H3N2) (55.3%) was the dominant subtype, followed by influenza B (30.9%; predominantly B/Yamagata‐lineage) and A(H1N1)pdm09 (13.9%). Circulation of influenza viruses began earlier in 2014 and 2015 than previously described, coincident with the emergence of A(H3N2) clades 3C.2a and 3C.3a, respectively. There was high diversity in the antigenicity of A(H3N2) viruses, and to a smaller extent influenza B viruses, during this period, with some mismatches with the northern and southern hemisphere vaccine formulations. All isolates tested were susceptible to the influenza antiviral drugs oseltamivir and zanamivir.

**Conclusions:**

Seasonal and year‐round co‐circulation of multiple influenza types/subtypes were detected in Cambodia during 2012‐2015.

## 1 BACKGROUND

Influenza viruses belong to the *Orthomyxoviridae* family of enveloped, segmented negative‐stranded RNA viruses. Currently, four antigenically distinct influenza viruses are responsible for human seasonal influenza infections, including two subtypes of influenza A [A(H1N1)pdm09 and A(H3N2)] and two lineages of influenza B (B/Yamagata and B/Victoria). Influenza is responsible for a large proportion of human morbidity and mortality as a result of infections worldwide. Annual influenza infections are estimated to result in approximately 3 to 5 million cases of severe illness globally,^1^ and 290 000 to 650 000 deaths.^2^

Seasonal influenza epidemics occur every year in temperate regions during the winter months^3^: November to March/April in the northern hemisphere and May to September in the southern hemisphere.^4^, ^5^ Influenza seasonality is more variable in tropical/subtropical regions where circulation can be observed year‐round, although activity is often more intense during rainy seasons.^6^ In addition, influenza activity is punctuated by occasional pandemics arising from the introduction of novel influenza A viruses into human circulation. These pandemics can significantly increase morbidity and mortality worldwide, with major economic impacts.^7^

We have previously described the circulation and seasonality of influenza viruses in Cambodia during six consecutive years (2006‐2011) following the establishment of the Cambodian National Influenza Centre (NIC) in 2006.^8^, ^9^, ^10^ These previous data demonstrated a peak in influenza circulation during the rainy season from June to November, which is consistent with influenza circulation in the southern hemisphere. However, year‐round circulation was also described, characteristic of influenza seasonality in tropical/subtropical regions, including some other Southeast Asian countries.^11^, ^12^ This current study furthers our understanding of influenza in Cambodia and describes the seasonal circulation, genetic and antigenic diversity, and antiviral drug susceptibility analyses of influenza viruses in Cambodia during four consecutive years (2012‐2015).

## 2 MATERIALS AND METHODS

### 2.1 Ethical statement

The Cambodian ILI surveillance system is a public health activity managed by the Ministry of Health in Cambodia and has a standing authorization from the National Ethics Committee for Human Research. Samples and patient information were anonymized for the purpose of this surveillance.

### 2.2 Geographic background

Cambodia is a tropical climate country in Southeast Asia with more than 15.5 million people, situated in the southwestern part of the Indochina peninsula and sharing international borders with Thailand and Laos on the West and North, and Vietnam on the East and Southeast.^13^ The country is affected by the Asian monsoon and is mostly hot and humid with a mean temperature of 27ºC and mean relative humidity of 77.5%. Similar to other subtropical/tropical areas, Cambodia has two distinct seasons: the dry season, which generally runs from November to April; and the rainy season, which starts in May‐June and ends in October‐November.

### 2.3 ILI surveillance system in Cambodia

The Cambodian National Influenza Center (NIC) was established in August 2006 at the Institute Pasteur in Cambodia (IPC). It is a joint collaboration between IPC, the Communicable Disease Control Department of the Ministry of Health (CDC/MoH), and the World Health Organization (WHO) office in Cambodia for documenting the dynamics of influenza disease and conducting virological characterization of circulating influenza strains.

**Figure 1:**
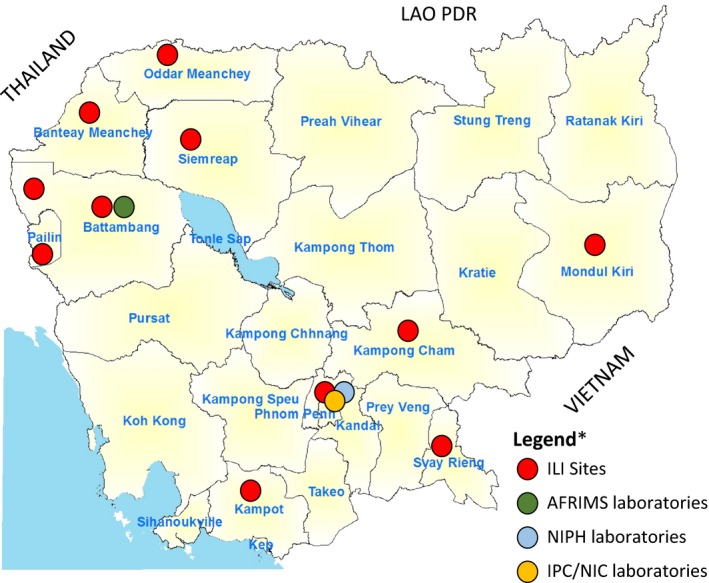
Map of Cambodia showing the influenza‐like illness sentinel surveillance sites and supporting laboratories. ILI, influenza‐like illness; AFRIMS, Armed Forces Research Institute of Medical Sciences; NIPH, National Institute of Public Health; IPC, Institute Pasteur in Cambodia; NIC, National Influenza Centre

An outpatient sentinel surveillance system for influenza‐like illness (ILI) with a weekly reporting and sampling scheme was established. Six ILI sentinel surveillance sites were operated in the referral hospitals of Kampong Cham (Eastern Cambodia), Mondulkiri (Eastern Cambodia), Svay Rieng (Southeast Cambodia), and Kampot (Southwest Cambodia) Provinces; and in a children's hospital in Siem Reap (northwestern Cambodia) and in the national pediatric hospital in Phnom Penh (capital city of Cambodia) by the CDC/MoH in collaboration with the National Institute of Public Health (NIPH), with assistance from the US Centers for Disease Control and Prevention and WHO Cambodia. The Armed Forces Research Institute of Medical Sciences (AFRIMS) operated an additional five sentinel sites in northwestern Cambodia: Thmor Kol Health Center (Battambang Province), Ta Sahn Health Center (Battambang Province), Anlong Veng Referral Hospital (Oddar Meanchey Province), Banteay Meanchey Health Center (Banteay Meanchey Province), and Preah Punlea Health Centre (Pailin Province). The 11 hospital sites included in the Cambodian outpatient surveillance system for ILI are presented in Figure 1.

The case definition for ILI was defined as previously described by WHO: sudden onset of fever (≥38ºC axillary temperature) and cough or sore throat in the absence of other diagnosis.^9^, ^14^

### 2.4 Specimen collection

Between January 2012 and December 2015, specimens and surveillance data were collected from a subset of outpatients presenting with ILI at sentinel surveillance sites. During 2012, ILI samples were collected as described previously.^9^ Starting in 2013 (and continuing through 2015), primary testing was shifted to the NIPH and AFRIMS laboratories, with the NIC concentrating on reference center activities. As such, during 2013‐2015 all ILI samples were first screened by the laboratories at NIPH or AFRIMS and then influenza‐positive samples (plus ~30% negative samples for quality control testing) were forwarded to the NIC for confirmation and viral characterization.

### 2.5 Laboratory methods

At the NIC, viral RNA was extracted using the QIAamp Viral RNA Mini Kit (Qiagen, CA, USA) and amplified using real‐time RT‐PCR to detect influenza A and B viruses using standard protocols. Influenza A viruses were subsequently subtyped using subtype‐specific real‐time reverse transcriptase polymerase chain reaction (RT‐qPCR) assays targeting H1pdm, H1, H3, H5, H7, N1pdm, N1, and N2 genes.^8^, ^9^ All influenza primers were sourced from the International Reagent Resource (https://www.internationalreagentresource.org/Home.aspx).

Influenza viruses were isolated at the IPC laboratory by inoculation of the specimens that tested positive by real‐time RT‐PCR onto Madin‐Darby canine kidney (MDCK) cells in an enhanced biosafety level 2 laboratory.^9^ The influenza isolates were characterized by hemagglutination inhibition assay (HAI) using reference antigens and anti‐sera provided by the WHO Collaborating Center (WHOCC) for Reference and Research on Influenza in Melbourne, Australia. A representative number of influenza isolates were sent each year to the WHOCC in Melbourne for confirmation and further analysis, including antiviral testing and partial or full genome sequencing of representative viruses.

### 2.6 Genome sequencing and phylogenetic analysis

Viral RNA extracted from MDCK supernatant was used to sequence the HA gene of all influenza isolates at the NIC laboratory using Sanger sequencing. At the WHOCC (Melbourne), a single‐reaction, multiplex RT‐PCR method that amplifies the HA, NA, and M genomic segments of seasonal influenza A and B viruses for next‐generation sequencing was used, as previously described.^15^ Nucleotide sequences from the coding regions of the HA genes of A(H3N2), A(H1N1)pdm09, and influenza B viruses were aligned using the Mafft multiple aligner V1.3.7 in the Geneious V10.0.9 software package (www.geneious.com). Sequences originating from Cambodia, surrounding countries, and representative reference sequences were downloaded from the EpiFlu™ Database (www.gisaid.org). Maximum likelihood trees were estimated using PhyML 3.0^16^ with 1000 bootstrap replicates using the ATGC server (http://www.atgc-montpellier.fr/phyml/execution). The most appropriate nucleotide substitution method determined for each data set was the GTR + G model.

The complete matrix gene was sequenced from representative influenza A viruses using previously described methods,^15^ to ascertain the presence of mutations (eg, Ser31Asn) associated with resistance to the adamantine class of inhibitors.

### 2.7 Nucleotide sequence accession numbers

All Cambodian influenza A(H3N2), A(H1N1)pdm09, and influenza B viral sequences included in the analysis were submitted to the EpiFlu™ Database, and all of these sequences are available via the GISAID website (https://www.gisaid.org/). Table S1 provides detailed information about all of the Cambodian isolates and sequences analyzed in this study.

### 2.8 Antiviral susceptibility testing

All influenza isolates sent each year to the WHOCC in Melbourne were analyzed for neuraminidase (NA) inhibitor susceptibility testing using an enzyme inhibition assay utilizing the fluorescent substrate MUNANA as described previously.^17^ The concentration of drug required to inhibit 50% of the NA activity (IC50) was calculated using the non‐linear curve fitting function in the GraphPad Prism 4 package (GraphPad Software). The average IC_50_ (nM) (± standard deviation) of two independent determinations was calculated for each virus. Outliers of more than two standard deviations from the overall mean were retested twice.^18^ Antiviral susceptibility was classified according to the guidelines from the WHO working group on surveillance of influenza antiviral susceptibility.^18^

### 2.9 Statistical analysis

The comparisons between percentages and two means were tested by chi‐squared (χ^2^) and Student's t test, respectively. A p value < 0.05 was considered statistically significant. Proportions, means, and all statistical analyses were performed using STATA 9.0 (Statacorp).

## 3 RESULTS

### 3.1 Influenza activity in Cambodia

During 2012‐2015, 3,222 specimens were submitted to the Cambodian NIC and analyzed as part of the ILI surveillance system (Table 1). Influenza virus was detected in 1,238 samples during this period: 324 in 2012, 335 in 2013, 263 in 2014, and 316 in 2015. Influenza A viruses (n = 856, 69.1%) were detected more frequently than influenza B viruses (n = 382, 30.9%). A(H1N1)pdm09 and A(H3N2) viruses constituted 20.1% (n = 172) and 79.9% (n = 684) of influenza A virus subtypes detected, respectively.

**Table 1:** The detection and isolation of influenza viruses associated with influenza‐like illness (ILI) in Cambodia during 2012‐2015

	Samples received by NIC	Influenza‐positive samples				Successful influenza isolation^a^, ^b^			Isolates sent to WHO CC		
		H1N1pdm09	H3N2	Influenza B	Total	H1N1pdm09	H3N2	Influenza B	H1N1pdm09	H3N2	Influenza B
2012	1907^c^	26	218	80	324	6	88	50	0	37	1
2013	467	91	24	220	335	63	9	154	55	7	51
2014	404	36	212	15	263	33	174	11	11	30	3
2015	444	19	230	67	316	13	123	61	7	21	10
Total	3222	172	684	382	1238	115	194	276	73	95	65

a Influenza viruses were isolated in Madin‐Darby canine kidney (MDCK) cells.

b Virus isolation was only attempted on samples with high viral load, as determined by RT‐qPCR CT values.

c During 2012 first‐line screening was conducted by the National Influenza Centre (NIC).

**Figure 2:**
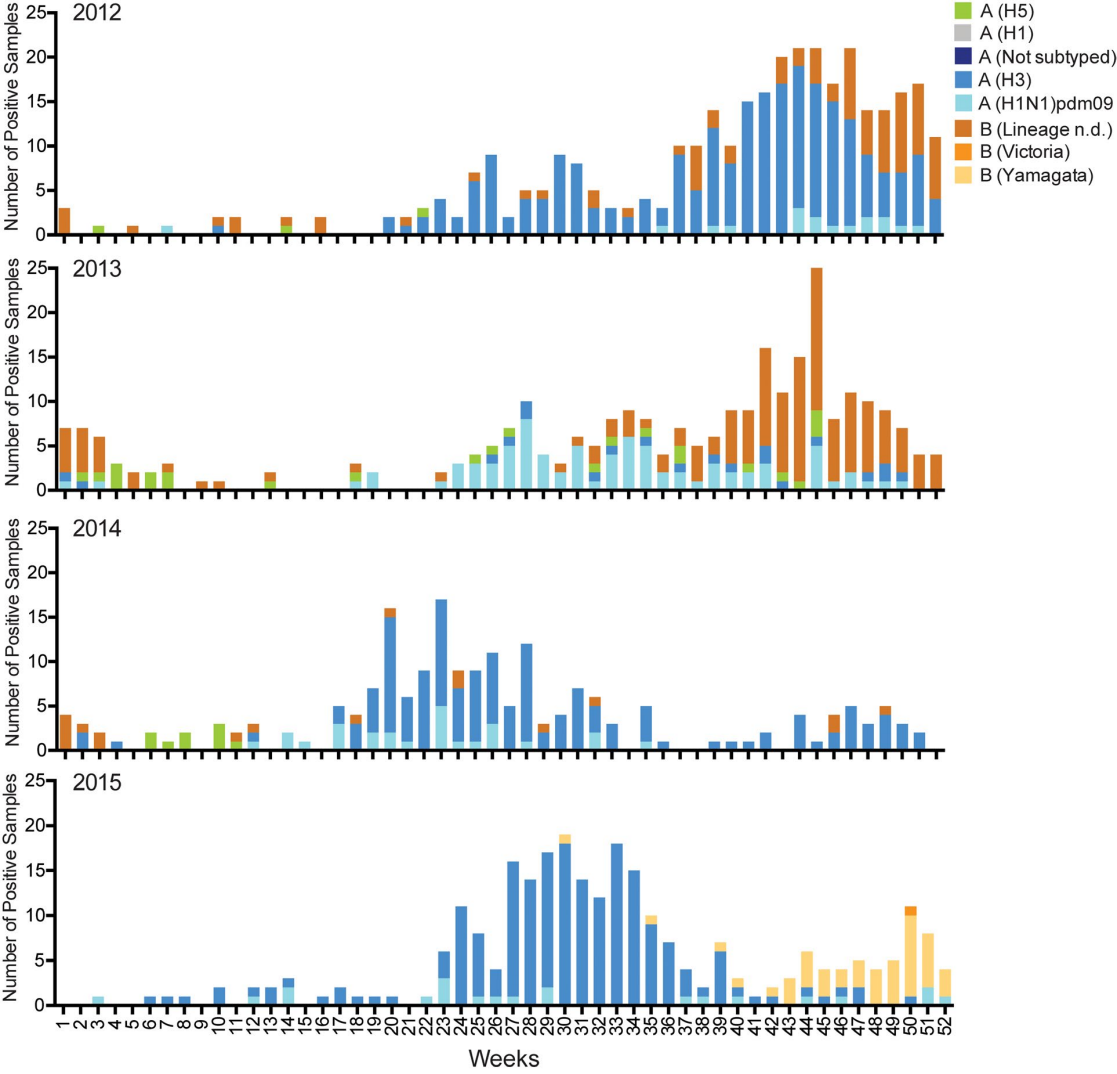
Number of specimens positive for influenza by subtype in Cambodia 2012‐2015 by week. Cambodian data accessed from the Global Initiative on Sharing All Influenza Data (GISAID—https://www.gisaid.org/)

Co‐circulation of A(H1N1)pdm09, A(H3N2), and influenza B viruses were detected across all four years, with A(H3N2) being the dominant subtype in 2012, 2014, and 2015; and influenza B the dominant virus in 2013. Both lineages of influenza B virus, B/Yamagata and B/Victoria, were detected across all four years, except in 2013 where only the B/Yamagata‐lineage was detected. From 2012 to 2015, influenza seasonality varied, with peak circulation occurring from September to December in 2012 and 2013; and from May to August in 2014 and 2015 (Figure 2).

Of the samples tested, the average age of influenza patients was 8.8 years (range, 4 days to 77 years) and 55.2% were male. The age and gender distribution of each year and across the four testing years (2012‐2015) for influenza patients are presented in Table S2.

### 3.2 Antigenic analysis

Generally, Cambodian seasonal influenza virus isolates matched the southern hemisphere trivalent inactivated influenza vaccine (TIIV) formulations during the relevant year they were released (Table 2). Antigenic analysis revealed that all of the A(H1N1)pdm09 isolates circulating in Cambodia during 2012‐2015 belonged to the A/California/7/2009(H1N1)‐like group. Mismatches occurred in 2012 when an A/Perth/16/2009(H3N2)‐like virus was included in both of the northern and southern hemisphere TIIVs, but A/Victoria/361/2011(H3N2)‐like viruses were the dominant circulating strains; also in 2012, a B/Brisbane/60/2008‐like virus was included in both of the northern and southern hemisphere TIIVs, but B/Wisconsin/1/2010‐like was the dominant strain; in 2014, the northern hemisphere formulation of TIIVs included an A/Victoria/361/2011(H3N2)‐like virus, but the dominant strain in Cambodia was A/Texas/50/2012(H3N2)‐like (which matched the southern hemisphere formulation); in 2015, an A/Texas/50/2012(H3N2)‐like virus was included in the northern hemisphere formulation of TIIVs, but A/Switzerland/9715293/2013(H3N2)‐like viruses were the dominant strains (which matched the southern hemisphere formulation); also in 2015, a B/Massachusetts/2/2012‐like was included in the northern hemisphere formulations of TIIVs, but the dominant circulating viruses were B/Phuket/3073/2013‐like (which matched the southern hemisphere formulation).

**Table 2:** The seasonal influenza strains circulating in Cambodia (2012‐2015) compared to the strains included in the WHO‐recommended vaccine formulations for trivalent influenza vaccines; viruses in bold indicate where the dominant Cambodian strain matched the vaccine strain

Year	Virus	Trivalent inactivated influenza vaccine strains		Cambodian circulating strains^b^ (proportion of Cambodian isolates)
		Northern hemisphere^a^	Southern hemisphere	
2012	A/H1N1pdm09	**A/California/7/2009‐like**	**A/California/7/2009‐like**	**A/California/7/2009‐like (100%)**
	A/H3N2	A/Perth/16/2009‐like	A/Perth/16/2009‐like	A/Victoria/361/2011‐like (91%) A/Perth/16/2009‐like (9%)
	Influenza B	B/Brisbane/60/2008‐like	B/Brisbane/60/2008‐like	B/Wisconsin/1/2010‐like (70%) B/Brisbane/60/2008‐like (30%)
2013	A/H1N1pdm09	**A/California/7/2009‐like**	**A/California/7/2009‐like**	**A/California/7/2009‐like (100%)**
	A/H3N2	**A/Victoria/361/2011‐like**	**A/Victoria/361/2011‐like**	**A/Victoria/361/2011‐like (100%)**
	Influenza B	**B/Wisconsin/1/2010‐like**	**B/Wisconsin/1/2010‐like**	**B/Wisconsin/1/2010‐like (52%)** B/Massachusetts/2/2012‐like (43%) B/Brisbane/60/2008‐like (5%)
2014	A/H1N1pdm09	**A/California/7/2009‐like**	**A/California/7/2009‐like**	**A/California/7/2009‐like (100%)**
	A/H3N2	A/Victoria/361/2011‐like	**A/Texas/50/2012‐like**	**A/Texas/50/2012‐like (69%)** A/Victoria/361/2011‐like (30%) A/Switzerland/9715293/2013‐like (1%)
	Influenza B	**B/Massachusetts/2/2012‐like**	**B/Massachusetts/2/2012‐like**	**B/Massachusetts/2/2012‐like (100%)**
2015	A/H1N1pdm09	**A/California/7/2009‐like**	**A/California/7/2009‐like**	**A/California/7/2009‐like (100%)**
	A/H3N2	A/Texas/50/2012‐like	**A/Switzerland/9715293/2013‐like**	**A/Switzerland/9715293/2013‐like** (76%) A/Hong Kong/4801/2014‐like (24%)
	Influenza B	B/Massachusetts/2/2012‐like	**B/Phuket/3073/2013‐like**	**B/Phuket/3073/2013‐like (96%)** B/Brisbane/60/2008‐like (4%)

a Northern Hemisphere vaccination periods occur mid‐year that is 2012 (2011/2012); 2013 (2012/2013); 2014 (2013/2014); 2015 (2014/2015).

b B/Brisbane/60/2008‐like viruses belong to the B/Victoria /7/87 lineage. B/Wisconsin/1/2010‐like viruses, B/Massachusetts/2/2012‐like viruses, and B/Phuket/3073/2013‐like viruses belong to the B/Yamagata/16/88 lineage.

### 3.3 Neuraminidase inhibitor susceptibility analysis

A total of 148 A(H3N2), 73 A(H1N1)pdm09, and 83 influenza B viruses were tested for susceptibility to the neuraminidase inhibitors oseltamivir and zanamivir. The analysis demonstrated that all of the tested isolates were sensitive to both drugs (Table S4). Full NA gene sequences were also generated for 68 A(H3N2), 25 A(H1N1)pdm09, and 48 influenza B viruses and confirmed that none contained mutations associated with NA inhibitor resistance (Table S3).

### 3.4 Sequence analysis of the matrix gene for mutations associated with amantadine resistance

Sequencing of the matrix gene was completed for representative A(H1N1)pdm09 (n = 27) and A(H3N2) (n = 66) viruses from 2012 to 2015. Sequence analysis showed that all of the Cambodian isolates contained an amino acid change from serine to asparagine at position 31 (Ser31Asn) in the M2 protein, which is associated with resistance to the adamantine class of inhibitors (Tables S4 and S5).

### 3.5 Phylogenetic analysis of A(H1N1)PDm09 Isolates

**Figure 3:**
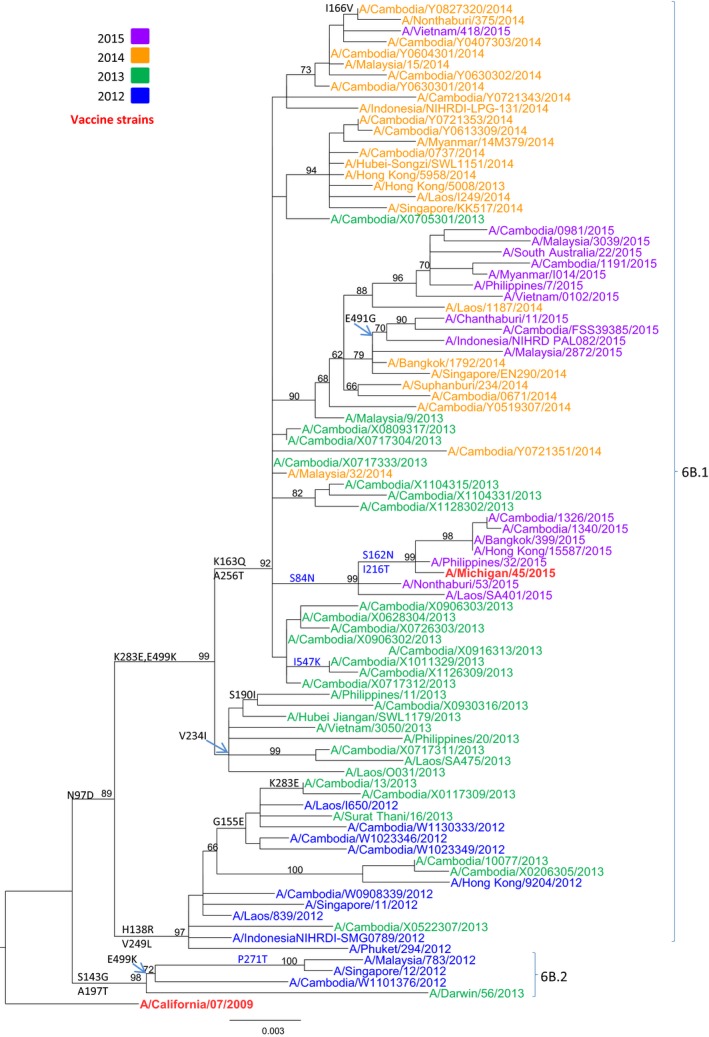
Phylogenetic analysis of the HA genes (sequences of 1650 nucleotides (nt49–1698)) of influenza A(H1N1)pdm09 virus isolates collected in Cambodia from 2012 to 2015. The phylogenetic analysis was conducted as a maximum likelihood phylogenetic tree of influenza using the GTR + G nucleotide substitution model with 1000 bootstrap replicates (values given on the branches) and rooted to A/California/07/2009. Major amino acid changes are shown in block letter at the appropriate nodes. Year of isolation is indicated by color: 2012—blue, 2013—green, 2014—yellow, 2015—purple. Vaccine strains are indicated in bold red. Other reference viruses are indicated in black. Scale bar indicates the number of nucleotide substitution per site

Phylogenetic analysis of the HA gene sequences was carried out for 70 representative Cambodian A(H1N1)pdm09 isolates from 2012 to 2015 (Figure 3; GISAID accession numbers are listed in Table S1). The HA sequences for clade reference strains (A/Darwin/56/2013, A/Michigan/45/2015, and A/South Australia/22/2015) and the vaccine strain (A/California/07/2009) were also included in the phylogenetic analysis. All Cambodian A(H1N1)pdm09 isolates clustered with clade 6B.1 viruses, except one isolate (A/Cambodia/W1101376/2012) which was isolated in 2012 and grouped with the reference strain A/Darwin/56/2013 in clade 6B.2. Interestingly, three Cambodian A(H1N1)pdm09 isolates from 2015 were grouped with the reference strain A/Michigan/45/2015 from clade 6B.1. All specific amino acid changes corresponding to each group of viruses are indicated in Figure 3.

### 3.6 Phylogenetic analysis of A(H3N2) isolates

**Figure 4:**
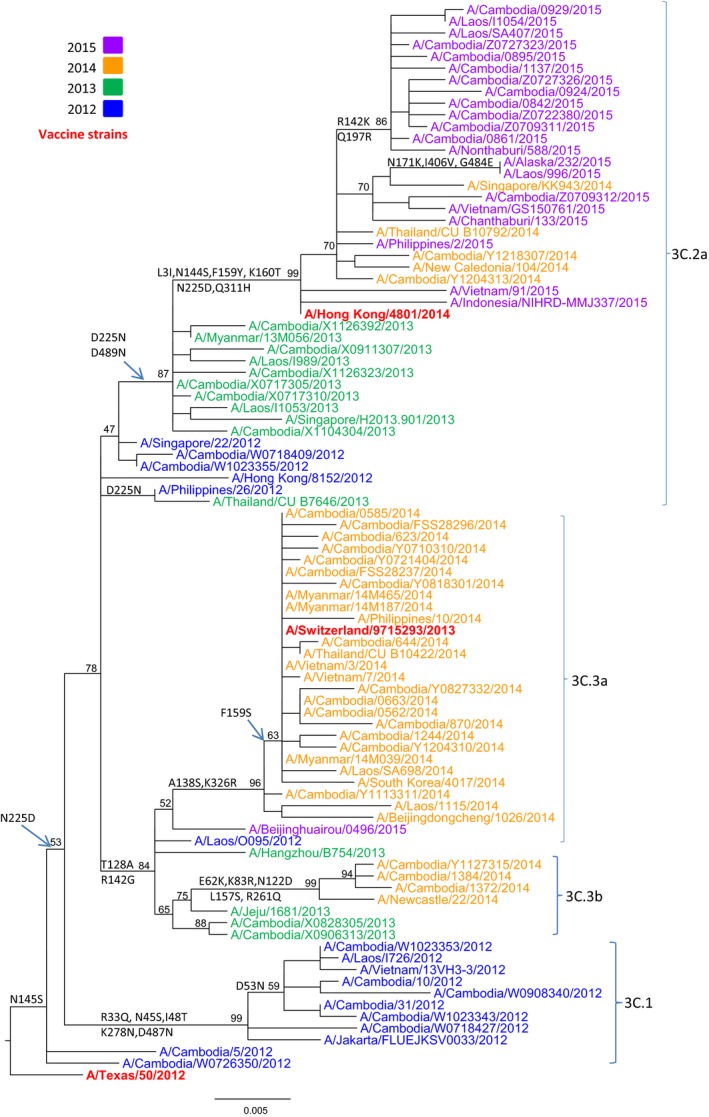
Phylogenetic analysis of the HA genes (sequences of 1653 nucleotides (nt49–1701) of influenza A(H3N2) virus isolates collected in Cambodia from 2012 to 2015. The phylogenetic analysis was conducted as a maximum likelihood phylogenetic tree of influenza using the GTR + G nucleotide substitution model with 1000 bootstrap replicates (values given on the branches) and rooted to A/Texas/50/2012. Major amino acid changes are shown in block letters at the appropriate nodes. Year of isolation is indicted by color: 2012—blue, 2013—green, 2014—yellow, 2015—purple. Vaccine strains are indicated in bold red. Other reference viruses are indicated in black. Scale bar indicates the number of nucleotide substitution per site

Phylogenetic analysis of the HA gene sequences was carried out for 108 representative A(H3N2) isolates from 2012 to 2015 in Cambodia. Additional reference sequences corresponding to vaccine candidate strains and A(H3N2) clade reference strains were included in the analysis. HA sequences of the A(H3N2) viruses isolated during the four consecutive seasons fell into four distinct clusters corresponding with each new influenza season (Figure 4; GISAID accession numbers are listed in Table S1): clade 3C.1 contained the majority of isolates from 2012; clade 3C.3b contained two viruses isolated in 2013 (A/Cambodia/X0828305/2013 and A/Cambodia/X0906313/2013) and some isolates from 2014; clade 3C.3a contained most of the isolates obtained in 2014; and clade 3C.2a contained two isolates from 2012 (A/Cambodia/W1023355/2012 and A/Cambodia/W0718409/2012), some isolates from 2013, two isolates from 2014 (A/Cambodia/Y1204313/2014 and A/Cambodia/Y1218307/2014), and all isolates obtained in 2015. The viruses in clade 3C.1 were closely related to the vaccine strain A/Texas/50/2012. The two isolates from 2013 and some isolates from 2014 that belonged to clade 3C.3b contained four more mutations compared to clade 3C.1. The Cambodian A(H3N2) viruses isolated in 2014 diverged into two clades. Some of the 2014 viruses belonged to clade 3C.3b with the reference A/Newcastle/22/2014 strain; however, the majority of isolates from 2014 were grouped with the vaccine strain A/Switzerland/9715293/2013, clade 3C.3a. The Cambodian A(H3N2) isolates belonging to clade 3C.2a were represented by the reference strain A/New Caledonia/104/2014 and the vaccine strain A/Hong Kong/4801/2014. All specific amino acid changes corresponding to each clade are indicated in Figure 4.

### 3.7 Phylogenetic analysis of influenza B isolates

**Figure 5:**
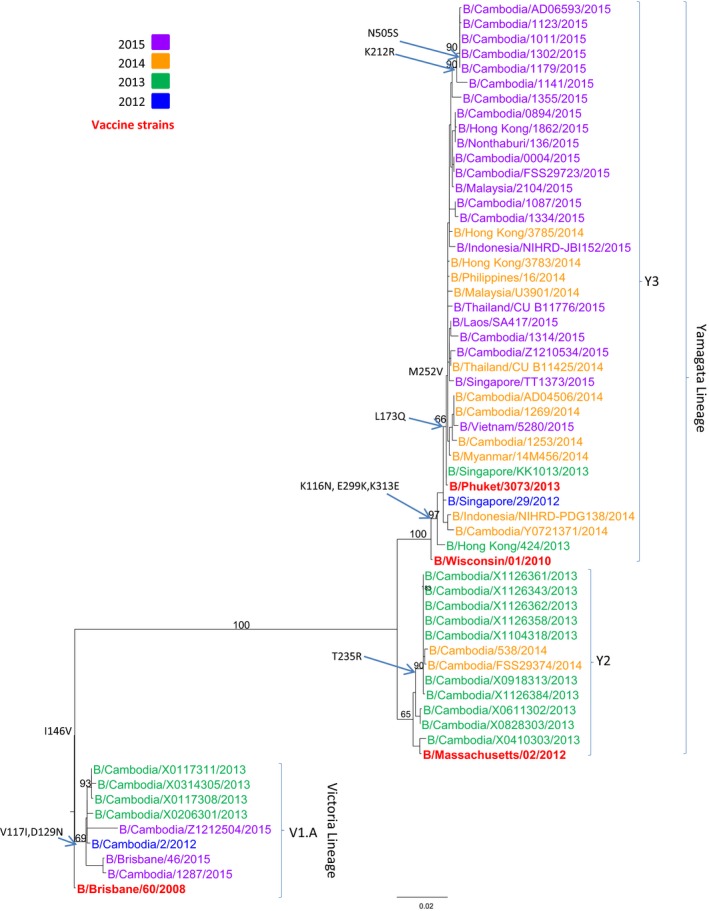
Phylogenetic analysis of the HA genes (sequences of 1710 nucleotides (nt 46–1755)) of influenza B virus isolates collected in Cambodia from 2012 to 2015. The phylogenetic analysis was conducted as a maximum likelihood phylogenetic tree of influenza using the GTR + G nucleotide substitution model with 1000 bootstrap replicates (values given on the branches) and rooted to B/Brisbane/60/2008. Major amino acid changes are shown in block letters at the appropriate nodes. Year of isolation is indicted by color: 2012—blue, 2013—green, 2014—yellow, 2015—purple. Vaccine strains are indicated in bold red. Other reference viruses are indicated in black, and the two major lineages B/Victoria‐like and B/Yamagata‐like are shown on the same figure. Scale bar indicates the number of nucleotide substitution per site

Phylogenetic analysis of the HA gene sequences was carried out for 56 representative Cambodian influenza B isolates from 2012 to 2015 (Figure 5; GISAID accession numbers and listed in Table S1). The Cambodian influenza B viruses were compared to the reference strains for B/Yamagata (B/Wisconsin/01/2010, B/Massachusetts/02/2012, and B/Phuket/3073/2013), and B/Victoria (B/Brisbane/60/2008) lineages. During this period, the majority of influenza B viruses circulating in Cambodia belonged to the B/Yamagata‐lineage. However, Cambodian influenza B isolates belonging to the B/Victoria‐lineage were also detected in 2012, 2013, and 2015. All of the Cambodian B/Victoria‐lineage strains clustered with B/Brisbane/46/2015 (clade V1.A). Two subgroups emerged within the Cambodian B/Yamagata‐lineage. One subgroup (B/Yamagata‐lineage, clade Y2), with most of the Cambodian isolates collected in 2013 and three isolates from 2014, was closely related to the vaccine strain B/Massachusetts/02/2012. The other subgroup of Cambodian influenza B/Yamagata‐lineage grouped with the vaccine strain B/Phuket/3073/2013 (B/Yamagata‐lineage, clade Y3), which included some of the isolates collected in 2014 and almost all isolates from 2015. All specific amino acid changes corresponding to each group of viruses are indicated in Figure 5.

## 4 DISCUSSION

During the surveillance period (2012‐2015), influenza circulation was dominated by A(H3N2), comprising 55% of the viruses detected, followed by influenza B (31%; predominantly B/Yamagata‐lineage) and A(H1N1)pdm09 (14%). These figures closely reflect influenza virus circulation during this period in the WHO Western Pacific Region, where A(H3N2) (51%), influenza B (32%), and A(H1N1)pdm09 (17%) were detected in similar proportions.^19^

Previous surveillance of influenza circulation in Cambodia from 2006‐2011^8^, ^9^, ^10^ revealed a consistent peak during the Cambodian rainy season from June to November, which broadly coincided with peak influenza circulation in the southern hemisphere. However, year‐round co‐circulation of multiple influenza subtypes reflected a similar pattern observed in many tropical countries. Hence, the Cambodian pattern of influenza transmission was postulated as intermediate between the temperate southern hemisphere and tropical countries.^9^ These general patterns were repeated in 2012‐2015. However, an earlier peak in influenza circulation was observed in 2014 (April‐August) and 2015 (June‐September), which coincided with the circulation of the clade 3C.2a and 3C.3a A(H3N2) viruses, the dominant viruses in these seasons, respectively. An unusual, earlier peak in seasonal influenza circulation, associated with A(H3N2) clade 3C.2a, was also observed in South Korea during 2016‐2017.^20^ There was also some evidence of bimodal circulation during 2014‐2015, particularly in 2015 when a second peak in influenza circulation was noted during October‐December, linked with an increase in influenza B cases. In all years included in the study, except 2014, influenza B viruses were primarily detected at the end of the influenza season, as influenza A cases were starting to wane. This pattern of influenza B circulation has previously been noted in Cambodia and other countries globally.^9^, ^21^ Seasonal influenza vaccination is not widespread in Cambodia, but considering the co‐circulation of both influenza B lineages in most years since surveillance began in 2006,^8^, ^9^ and the frequent mismatch between strains included in the TIIVs and the most dominant circulating influenza B lineage, the introduction of the quadrivalent seasonal influenza vaccine might be worthwhile, especially in young children, as this covers both lineages of influenza B virus and both subtypes of seasonal influenza A viruses.^22^

Antigenic characterization of Cambodian influenza isolates from 2012 to 2015 largely corresponded with the southern and northern hemisphere vaccine formulations. Vaccination for seasonal influenza is still rare in Cambodia, with vaccines generally only available through private clinics.^9^, ^23^ Future policies for the introduction of seasonal vaccination in Cambodia will be aided by the distinct seasonality of influenza circulation, which is unusual for a tropical country, and should be aligned with a vaccination program mirroring the WHO southern hemisphere vaccination formulation timetable with vaccinations ideally taking place in March‐April. Recent introduction of seasonal alert thresholds in Cambodia^24^ using the “WHO method”^25^ will also help to refine the ideal vaccination timing.

There was no detection of seasonal influenza isolates with resistance to the commonly used antivirals oseltamivir and zanamivir in Cambodia during 2012‐2015. These antivirals are generally not used in Cambodia during seasonal influenza infections, so resistance is unlikely to be affected by domestic antiviral use. Following the widespread reporting of adamantane resistance in A(H3N2) strains from 2005 and the emergence of the A(H1N1)pdm09 virus (which was already adamantane‐resistant), this antiviral is no longer recommended for use.^26^, ^27^ Sequence analysis of the matrix gene from representative Cambodian isolates suggested that resistance to adamantanes is common, as all isolates obtained during this period contained the Ser31Asn mutation in the M2 protein. This situation is still reflected currently with most isolates globally being resistant,^27^ despite the almost complete cessation in the use of adamantanes.

Samples from patients with ILI were collected through two different surveillance systems (managed by the NIPH and AFRIMS laboratories), leading to limitations in the analyses presented in this paper. However, any possible biases in the representativeness of viruses are likely countered by the large number of samples collected throughout the surveillance period and the large number of sentinel sites, covering most of the country. Individual patient data are compiled at the national level by the Cambodian Ministry of Health and was not analyzed by the Cambodian NIC. As such, epidemiological information such as disease severity and infection rates could not be included in these analyses. The large number of influenza‐positive samples (n = 1,238) received by the NIC during this period meant that only representative isolates could be included for genetic and antigenic characterization, meaning that some isolates with interesting characteristics could have been missed. Despite these limitations, we believe that the results are representative of the circulation and genetic/antigenic/antiviral characteristics of seasonal influenza strains in Cambodia during 2012‐2015.

Our data concur with the findings from previous studies describing the seasonal circulation of influenza viruses in Cambodia with year‐round co‐circulation of multiple influenza subtypes.^8^, ^9^, ^10^, ^24^ Influenza viruses detected in Cambodia continued to be susceptible to oseltamivir and zanamivir, but resistance to adamantanes was still universal in influenza A viruses. Antigenically, there was considerable drift in the A(H3N2) viruses and some in the influenza B viruses (B/Yamagata‐lineage viruses more than B/Victoria‐lineage viruses) during 2012‐2015, but little change in A(H1N1)pdm09 viruses. Seasonal influenza surveillance has been strengthened in Cambodia since being established in 2006 and continues to contribute to our knowledge of the regional and global circulation of seasonal influenza strains.

## 5 DISCLAIMER

Material has been reviewed by the Walter Reed Army Institute of Research. There is no objection to its presentation and/or publication. The opinions or assertions contained herein are the private views of the author and are not to be construed as official, or as reflecting true views of the Department of the Army or the Department of Defense. The investigators have adhered to the policies for protection of human subjects as prescribed in AR 70–25.
