# Supplementary material for: Circulation and characterization of seasonal influenza viruses in Cambodia, 2012‐2015
Source: Influenza Other Respir Viruses. 2019 Jun 28;13(5):465–76. doi: 10.1111/irv.12647 (PMC6692578; doi:10.1111/irv.12647)
Supplement: Supplementary file 1 [file IRV-13-465-s001.docx]

**Supplementary Table 1.** Accession numbers from the Cambodian influenza A/H3N2, A/H1N1pdm09 and influenza B virus hemagglutinin sequences included in the analyses (available via GISAID website: [www.gisaid.org](http://www.gisaid.org))

| **GISAID Accession#** | **Segment** | **Country** | **Collection date** | **Isolate name** |
| --- | --- | --- | --- | --- |
| EPI394250 | HA | Cambodia | 2012-Jul-04 | A/Cambodia/5/2012 |
| EPI394253 | HA | Cambodia | 2012-Jul-19 | A/Cambodia/10/2012 |
| EPI394322 | HA | Cambodia | 2012-Jun-27 | A/Cambodia/31/2012 |
| EPI872930 | HA | Cambodia | 2012-Jul-04 | A/Cambodia/W0711366/2012 |
| EPI872931 | HA | Cambodia | 2012-Jul-11 | A/Cambodia/W0718409/2012 |
| EPI872932 | HA | Cambodia | 2012-Jul-11 | A/Cambodia/W0718427/2012 |
| EPI872933 | HA | Cambodia | 2012-Jul-18 | A/Cambodia/W0726350/2012 |
| EPI743828 | HA | Cambodia | 2012-Aug-28 | A/Cambodia/W0908340/2012 |
| EPI743833 | HA | Cambodia | 2012-Sep-12 | A/Cambodia/W0921311/2012 |
| EPI743838 | HA | Cambodia | 2012-Oct-05 | A/Cambodia/W1023343/2012 |
| EPI743843 | HA | Cambodia | 2012-Oct-10 | A/Cambodia/W1023347/2012 |
| EPI743848 | HA | Cambodia | 2012-Oct-18 | A/Cambodia/W1023353/2012 |
| EPI743853 | HA | Cambodia | 2012-Oct-18 | A/Cambodia/W1023355/2012 |
| EPI872934 | HA | Cambodia | 2013-Jul-02 | A/Cambodia/X0717305/2013 |
| EPI491283 | HA | Cambodia | 2013-Jul-09 | A/Cambodia/X0717310/2013 |
| EPI872935 | HA | Cambodia | 2013-Aug-09 | A/Cambodia/X0828305/2013 |
| EPI872936 | HA | Cambodia | 2013-Aug-21 | A/Cambodia/X0906313/2013 |
| EPI872937 | HA | Cambodia | 2013-Aug-25 | A/Cambodia/X0911307/2013 |
| EPI872938 | HA | Cambodia | 2013-Sep-25 | A/Cambodia/X1011313/2013 |
| EPI872939 | HA | Cambodia | 2013-Sep-23 | A/Cambodia/X1011315/2013 |
| EPI872940 | HA | Cambodia | 2013-Oct-07 | A/Cambodia/X1104304/2013 |
| EPI872941 | HA | Cambodia | 2013-Oct-28 | A/Cambodia/X1126323/2013 |
| EPI872942 | HA | Cambodia | 2013-Oct-28 | A/Cambodia/X1126392/2013 |
| EPI540059 | HA | Cambodia | 2014-May-07 | A/Cambodia/0562/2014 |
| EPI540056 | HA | Cambodia | 2014-May-17 | A/Cambodia/0585/2014 |
| EPI540001 | HA | Cambodia | 2014-May-14 | A/Cambodia/590/2014 |
| EPI539997 | HA | Cambodia | 2014-May-19 | A/Cambodia/594/2014 |
| EPI540063 | HA | Cambodia | 2014-May-29 | A/Cambodia/615/2014 |
| EPI540065 | HA | Cambodia | 2014-May-26 | A/Cambodia/623/2014 |
| EPI540062 | HA | Cambodia | 2014-Jun-01 | A/Cambodia/644/2014 |
| EPI541600 | HA | Cambodia | 2014-Jun-02 | A/Cambodia/653/2014 |
| EPI540067 | HA | Cambodia | 2014-Jun-04 | A/Cambodia/0658/2014 |
| EPI540051 | HA | Cambodia | 2014-Jun-05 | A/Cambodia/0663/2014 |
| EPI539996 | HA | Cambodia | 2014-Jun-19 | A/Cambodia/712/2014 |
| EPI539993 | HA | Cambodia | 2014-Jun-18 | A/Cambodia/715/2014 |
| EPI539994 | HA | Cambodia | 2014-Jun-26 | A/Cambodia/735/2014 |
| EPI539995 | HA | Cambodia | 2014-Jun-26 | A/Cambodia/736/2014 |
| EPI578858 | HA | Cambodia | 2014-Aug-05 | A/Cambodia/870/2014 |
| EPI579366 | HA | Cambodia | 2014-Nov-11 | A/Cambodia/1244/2014 |
| EPI575344 | HA | Cambodia | 2014-Dec-11 | A/Cambodia/1370/2014 |
| EPI575352 | HA | Cambodia | 2014-Dec-11 | A/Cambodia/1372/2014 |
| EPI575158 | HA | Cambodia | 2014-Dec-17 | A/Cambodia/1384/2014 |
| EPI612262 | HA | Cambodia | 2014-Dec-13 | A/Cambodia/1393/2014 |
| EPI629750 | HA | Cambodia | 2014-Oct-18 | A/Cambodia/AD04410/2014 |
| EPI629753 | HA | Cambodia | 2014-Nov-12 | A/Cambodia/AD04526/2014 |
| EPI551518 | HA | Cambodia | 2014-Jun-16 | A/Cambodia/FSS28237/2014 |
| EPI629756 | HA | Cambodia | 2014-Sep-30 | A/Cambodia/FSS28296/2014 |
| EPI872943 | HA | Cambodia | 2014-May-10 | A/Cambodia/Y0526301/2014 |
| EPI872944 | HA | Cambodia | 2014-May-23 | A/Cambodia/Y0529312/2014 |
| EPI872945 | HA | Cambodia | 2014-Jun-12 | A/Cambodia/Y0616341/2014 |
| EPI551525 | HA | Cambodia | 2014-Jun-19 | A/Cambodia/Y0703302/2014 |
| EPI872946 | HA | Cambodia | 2014-Jun-23 | A/Cambodia/Y0710306/2014 |
| EPI872947 | HA | Cambodia | 2014-Jun-25 | A/Cambodia/Y0710310/2014 |
| EPI551530 | HA | Cambodia | 2014-May-28 | A/Cambodia/Y0721397/2014 |
| EPI872948 | HA | Cambodia | 2014-Jun-03 | A/Cambodia/Y0721404/2014 |
| EPI872949 | HA | Cambodia | 2014-Jun-12 | A/Cambodia/Y0721427/2014 |
| EPI872950 | HA | Cambodia | 2014-Jun-20 | A/Cambodia/Y0721438/2014 |
| EPI551535 | HA | Cambodia | 2014-Jun-26 | A/Cambodia/Y0721447/2014 |
| EPI872959 | HA | Cambodia | 2014-Jul-14 | A/Cambodia/Y0811312/2014 |
| EPI872960 | HA | Cambodia | 2014-Jul-15 | A/Cambodia/Y0813303/2014 |
| EPI872961 | HA | Cambodia | 2014-Aug-05 | A/Cambodia/Y0818301/2014 |
| EPI872962 | HA | Cambodia | 2014-Aug-15 | A/Cambodia/Y0827331/2014 |
| EPI872963 | HA | Cambodia | 2014-Aug-08 | A/Cambodia/Y0827332/2014 |
| EPI873553 | HA | Cambodia | 2014-Oct-30 | A/Cambodia/Y1113311/2014 |
| EPI873020 | HA | Cambodia | 2014-Oct-31 | A/Cambodia/Y1127315/2014 |
| EPI873021 | HA | Cambodia | 2014-Nov-11 | A/Cambodia/Y1127320/2014 |
| EPI629759 | HA | Cambodia | 2014-Nov-26 | A/Cambodia/Y1204310/2014 |
| EPI873022 | HA | Cambodia | 2014-Nov-28 | A/Cambodia/Y1204313/2014 |
| EPI629762 | HA | Cambodia | 2014-Dec-02 | A/Cambodia/Y1218307/2014 |
| EPI746659 | HA | Cambodia | 2015-Sep-22 | A/Cambodia/0009/2015 |
| EPI711282 | HA | Cambodia | 2015-Aug-05 | A/Cambodia/0840/2015 |
| EPI711290 | HA | Cambodia | 2015-Aug-07 | A/Cambodia/0842/2015 |
| EPI711298 | HA | Cambodia | 2015-Aug-13 | A/Cambodia/0861/2015 |
| EPI712418 | HA | Cambodia | 2015-Aug-14 | A/Cambodia/0869/2015 |
| EPI715253 | HA | Cambodia | 2015-Aug-18 | A/Cambodia/0877/2015 |
| EPI712426 | HA | Cambodia | 2015-Aug-24 | A/Cambodia/0887/2015 |
| EPI711306 | HA | Cambodia | 2015-Aug-25 | A/Cambodia/0895/2015 |
| EPI712434 | HA | Cambodia | 2015-Aug-26 | A/Cambodia/0909/2015 |
| EPI702135 | HA | Cambodia | 2015-Aug-27 | A/Cambodia/0911/2015 |
| EPI711314 | HA | Cambodia | 2015-Aug-26 | A/Cambodia/0917/2015 |
| EPI702040 | HA | Cambodia | 2015-Aug-31 | A/Cambodia/0924/2015 |
| EPI711019 | HA | Cambodia | 2015-Sep-01 | A/Cambodia/0929/2015 |
| EPI711322 | HA | Cambodia | 2015-Sep-02 | A/Cambodia/0942/2015 |
| EPI711330 | HA | Cambodia | 2015-Sep-08 | A/Cambodia/0951/2015 |
| EPI711027 | HA | Cambodia | 2015-Oct-28 | A/Cambodia/1137/2015 |
| EPI730018 | HA | Cambodia | 2015-Nov-11 | A/Cambodia/1181/2015 |
| EPI727326 | HA | Cambodia | 2015-Nov-11 | A/Cambodia/1201/2015 |
| EPI727337 | HA | Cambodia | 2015-Dec-03 | A/Cambodia/1290/2015 |
| EPI676125 | HA | Cambodia | 2015-Jun-18 | A/Cambodia/Ad05096/2015 |
| EPI652592 | HA | Cambodia | 2015-Jun-24 | A/Cambodia/Ad05323/2015 |
| EPI765131 | HA | Cambodia | 2015-Dec-08 | A/Cambodia/FSS31758/2015 |
| EPI652613 | HA | Cambodia | 2015-Jul-01 | A/Cambodia/Z0709310/2015 |
| EPI648839 | HA | Cambodia | 2015-Jun-26 | A/Cambodia/Z0709311/2015 |
| EPI676122 | HA | Cambodia | 2015-Jun-29 | A/Cambodia/Z0709312/2015 |
| EPI652595 | HA | Cambodia | 2015-Jul-02 | A/Cambodia/Z0709313/2015 |
| EPI652598 | HA | Cambodia | 2015-Jul-14 | A/Cambodia/Z0722377/2015 |
| EPI652580 | HA | Cambodia | 2015-Jul-15 | A/Cambodia/Z0722378/2015 |
| EPI652601 | HA | Cambodia | 2015-Jul-15 | A/Cambodia/Z0722379/2015 |
| EPI652604 | HA | Cambodia | 2015-Jul-14 | A/Cambodia/Z0722380/2015 |
| EPI652616 | HA | Cambodia | 2015-Jul-01 | A/Cambodia/Z0722381/2015 |
| EPI652571 | HA | Cambodia | 2015-Jun-11 | A/Cambodia/Z0727320/2015 |
| EPI676119 | HA | Cambodia | 2015-Jun-24 | A/Cambodia/Z0727323/2015 |
| EPI652583 | HA | Cambodia | 2015-Jun-28 | A/Cambodia/Z0727325/2015 |
| EPI652574 | HA | Cambodia | 2015-Jun-25 | A/Cambodia/Z0727326/2015 |
| EPI676116 | HA | Cambodia | 2015-May-26 | A/Cambodia/Z0727327/2015 |
| EPI652607 | HA | Cambodia | 2015-Jun-29 | A/Cambodia/Z0727328/2015 |
| EPI652610 | HA | Cambodia | 2015-Jun-29 | A/Cambodia/Z0727329/2015 |
| EPI652577 | HA | Cambodia | 2015-Jun-29 | A/Cambodia/Z0727330/2015 |
| EPI648842 | HA | Cambodia | 2015-Jun-08 | A/Cambodia/Z0727331/2015 |
| EPI873217 | HA | Cambodia | 2012-Jul-18 | A/Cambodia/W0908339/2012 |
| EPI873023 | HA | Cambodia | 2012-Oct-08 | A/Cambodia/W1023346/2012 |
| EPI873024 | HA | Cambodia | 2012-Oct-11 | A/Cambodia/W1023349/2012 |
| EPI873025 | HA | Cambodia | 2012-Oct-19 | A/Cambodia/W1023356/2012 |
| EPI873026 | HA | Cambodia | 2012-Sep-07 | A/Cambodia/W1101376/2012 |
| EPI873211 | HA | Cambodia | 2012-Nov-14 | A/Cambodia/W1130333/2012 |
| EPI443611 | HA | Cambodia | 2013-Jan-04 | A/Cambodia/13/2013 |
| EPI443614 | HA | Cambodia | 2013-Jan-23 | A/Cambodia/10077/2013 |
| EPI873214 | HA | Cambodia | 2013-Jan-02 | A/Cambodia/X0117309/2013 |
| EPI491259 | HA | Cambodia | 2013-Jan-22 | A/Cambodia/X0206305/2013 |
| EPI491262 | HA | Cambodia | 2013-May-22 | A/Cambodia/X0522305/2013 |
| EPI873215 | HA | Cambodia | 2015-May-22 | A/Cambodia/X0522307/2013 |
| EPI873216 | HA | Cambodia | 2013-Jun-25 | A/Cambodia/X0628304/2013 |
| EPI873218 | HA | Cambodia | 2013-Jun-25 | A/Cambodia/X0628307/2013 |
| EPI873219 | HA | Cambodia | 2013-Jun-21 | A/Cambodia/X0705301/2013 |
| EPI491289 | HA | Cambodia | 2013-Jun-22 | A/Cambodia/X0717301/2013 |
| EPI873220 | HA | Cambodia | 2013-Jul-02 | A/Cambodia/X0717303/2013 |
| EPI873221 | HA | Cambodia | 2013-Jun-27 | A/Cambodia/X0717304/2013 |
| EPI873222 | HA | Cambodia | 2013-Jul-03 | A/Cambodia/X0717311/2013 |
| EPI491542 | HA | Cambodia | 2013-Jul-03 | A/Cambodia/X0717312/2013 |
| EPI491292 | HA | Cambodia | 2013-Jul-12 | A/Cambodia/X0717333/2013 |
| EPI873223 | HA | Cambodia | 2013-Jul-10 | A/Cambodia/X0726303/2013 |
| EPI873224 | HA | Cambodia | 2013-Aug-19 | A/Cambodia/X0821311/2013 |
| EPI873225 | HA | Cambodia | 2013-Jul-23 | A/Cambodia/X0809317/2013 |
| EPI873226 | HA | Cambodia | 2013-Aug-06 | A/Cambodia/X0816311/2013 |
| EPI873227 | HA | Cambodia | 2013-Aug-05 | A/Cambodia/X0816320/2013 |
| EPI873228 | HA | Cambodia | 2013-Aug-14 | A/Cambodia/X0906302/2013 |
| EPI873229 | HA | Cambodia | 2013-Aug-28 | A/Cambodia/X0916306/2013 |
| EPI873230 | HA | Cambodia | 2013-Aug-30 | A/Cambodia/X0916311/2013 |
| EPI873231 | HA | Cambodia | 2013-Sep-04 | A/Cambodia/X0916313/2013 |
| EPI529447 | HA | Cambodia | 2013-Sep-13 | A/Cambodia/X0930306/2013 |
| EPI873232 | HA | Cambodia | 2013-Sep-19 | A/Cambodia/X0930316/2013 |
| EPI873233 | HA | Cambodia | 2013-Sep-05 | A/Cambodia/X0918311/2013 |
| EPI873234 | HA | Cambodia | 2013-Aug-14 | A/Cambodia/X0906303/2013 |
| EPI873235 | HA | Cambodia | 2013-Sep-24 | A/Cambodia/X0926312/2013 |
| EPI873301 | HA | Cambodia | 2013-Sep-28 | A/Cambodia/X1009301/2013 |
| EPI873570 | HA | Cambodia | 2013-Sep-25 | A/Cambodia/X1011329/2013 |
| EPI529450 | HA | Cambodia | 2013-Oct-09 | A/Cambodia/X1104315/2013 |
| EPI873303 | HA | Cambodia | 2013-Oct-24 | A/Cambodia/X1104331/2013 |
| EPI873380 | HA | Cambodia | 2013-Oct-16 | A/Cambodia/X1126309/2013 |
| EPI873537 | HA | Cambodia | 2013-Nov-04 | A/Cambodia/X1126400/2013 |
| EPI873538 | HA | Cambodia | 2013-Nov-28 | A/Cambodia/X1128302/2013 |
| EPI541527 | HA | Cambodia | 2014-Jun-04 | A/Cambodia/0671/2014 |
| EPI541533 | HA | Cambodia | 2014-Jun-26 | A/Cambodia/0737/2014 |
| EPI541530 | HA | Cambodia | 2014-Jun-26 | A/Cambodia/0745/2014 |
| EPI873539 | HA | Cambodia | 2014-Apr-05 | A/Cambodia/Y0407303/2014 |
| EPI873540 | HA | Cambodia | 2014-May-02 | A/Cambodia/Y0519307/2014 |
| EPI873541 | HA | Cambodia | 2014-May-31 | A/Cambodia/Y0604301/2014 |
| EPI873542 | HA | Cambodia | 2014-Jun-06 | A/Cambodia/Y0630301/2014 |
| EPI873543 | HA | Cambodia | 2014-May-29 | A/Cambodia/Y0613309/2014 |
| EPI551356 | HA | Cambodia | 2014-Jun-11 | A/Cambodia/Y0630302/2014 |
| EPI873544 | HA | Cambodia | 2014-Jun-04 | A/Cambodia/Y0613313/2014 |
| EPI873545 | HA | Cambodia | 2014-Apr-07 | A/Cambodia/Y0721343/2014 |
| EPI873546 | HA | Cambodia | 2014-Apr-11 | A/Cambodia/Y0721347/2014 |
| EPI873547 | HA | Cambodia | 2014-Apr-24 | A/Cambodia/Y0721351/2014 |
| EPI873548 | HA | Cambodia | 2014-Apr-28 | A/Cambodia/Y0721353/2014 |
| EPI873549 | HA | Cambodia | 2014-May-08 | A/Cambodia/Y0721368/2014 |
| EPI873550 | HA | Cambodia | 2014-May-16 | A/Cambodia/Y0721377/2014 |
| EPI873551 | HA | Cambodia | 2014-Jun-04 | A/Cambodia/Y0721417/2014 |
| EPI551359 | HA | Cambodia | 2014-Jun-26 | A/Cambodia/Y0721444/2014 |
| EPI873552 | HA | Cambodia | 2014-Aug-07 | A/Cambodia/Y0827320/2014 |
| EPI565259 | HA | Cambodia | 2014-Dec-05 | A/Cambodia/Y1218309/2014 |
| EPI697810 | HA | Cambodia | 2015-Sep-12 | A/Cambodia/0981/2015 |
| EPI697818 | HA | Cambodia | 2015-Sep-25 | A/Cambodia/1021/2015 |
| EPI759328 | HA | Cambodia | 2015-Nov-05 | A/Cambodia/1191/2015 |
| EPI759336 | HA | Cambodia | 2015-Dec-09 | A/Cambodia/1300/2015 |
| EPI759344 | HA | Cambodia | 2015-Dec-15 | A/Cambodia/1326/2015 |
| EPI759352 | HA | Cambodia | 2015-Dec-21 | A/Cambodia/1340/2015 |
| EPI636078 | HA | Cambodia | 2015-Jun-16 | A/Cambodia/FSS39385/2015 |
| EPI765117 | HA | Cambodia | 2015-Sep-25 | A/Cambodia/Z1210510/2015 |
| EPI417348 | HA | Cambodia | 2012-Apr-11 | B/Cambodia/2/2012 |
| EPI873554 | HA | Cambodia | 2013-Jan-04 | B/Cambodia/X0117308/2013 |
| EPI873555 | HA | Cambodia | 2013-Jan-02 | B/Cambodia/X0117311/2013 |
| EPI873556 | HA | Cambodia | 2013-Jan-16 | B/Cambodia/X0206301/2013 |
| EPI873557 | HA | Cambodia | 2013-Mar-12 | B/Cambodia/X0314305/2013 |
| EPI873558 | HA | Cambodia | 2013-Apr-10 | B/Cambodia/X0410303/2013 |
| EPI491277 | HA | Cambodia | 2013-May-29 | B/Cambodia/X0611302/2013 |
| EPI873559 | HA | Cambodia | 2013-Aug-08 | B/Cambodia/X0828303/2013 |
| EPI873560 | HA | Cambodia | 2013-Aug-19 | B/Cambodia/X0906306/2013 |
| EPI873561 | HA | Cambodia | 2013-Sep-06 | B/Cambodia/X0918313/2013 |
| EPI873562 | HA | Cambodia | 2013-Oct-09 | B/Cambodia/X1022309/2013 |
| EPI873563 | HA | Cambodia | 2013-Oct-09 | B/Cambodia/X1104318/2013 |
| EPI873564 | HA | Cambodia | 2013-Oct-30 | B/Cambodia/X1126335/2013 |
| EPI873565 | HA | Cambodia | 2013-Oct-30 | B/Cambodia/X1126338/2013 |
| EPI873566 | HA | Cambodia | 2013-Nov-05 | B/Cambodia/X1126343/2013 |
| EPI873567 | HA | Cambodia | 2013-Nov-12 | B/Cambodia/X1126358/2013 |
| EPI529383 | HA | Cambodia | 2013-Nov-13 | B/Cambodia/X1126361/2013 |
| EPI873568 | HA | Cambodia | 2013-Nov-12 | B/Cambodia/X1126362/2013 |
| EPI873569 | HA | Cambodia | 2013-Oct-31 | B/Cambodia/X1126369/2013 |
| EPI529386 | HA | Cambodia | 2013-Nov-25 | B/Cambodia/X1126384/2013 |
| EPI540560 | HA | Cambodia | 2014-May-03 | B/Cambodia/538/2014 |
| EPI620355 | HA | Cambodia | 2014-Nov-13 | B/Cambodia/1253/2014 |
| EPI582432 | HA | Cambodia | 2014-Nov-17 | B/Cambodia/1269/2014 |
| EPI630028 | HA | Cambodia | 2014-Nov-02 | B/Cambodia/AD04506/2014 |
| EPI562021 | HA | Cambodia | 2014-Nov-11 | B/Cambodia/Fss26970/2014 |
| EPI562024 | HA | Cambodia | 2014-Dec-08 | B/Cambodia/FSS29374/2014 |
| EPI551252 | HA | Cambodia | 2014-May-19 | B/Cambodia/Y0721371/2014 |
| EPI562027 | HA | Cambodia | 2014-Aug-09 | B/Cambodia/Y0827324/2014 |
| EPI765309 | HA | Cambodia | 2015-Dec-30 | B/Cambodia/0004/2015 |
| EPI696757 | HA | Cambodia | 2015-Aug-21 | B/Cambodia/0894/2015 |
| EPI696765 | HA | Cambodia | 2015-Sep-23 | B/Cambodia/1011/2015 |
| EPI696773 | HA | Cambodia | 2015-Oct-07 | B/Cambodia/1072/2015 |
| EPI696781 | HA | Cambodia | 2015-Oct-06 | B/Cambodia/1087/2015 |
| EPI696789 | HA | Cambodia | 2015-Oct-21 | B/Cambodia/1122/2015 |
| EPI696797 | HA | Cambodia | 2015-Oct-22 | B/Cambodia/1123/2015 |
| EPI696805 | HA | Cambodia | 2015-Oct-27 | B/Cambodia/1141/2015 |
| EPI763144 | HA | Cambodia | 2015-Nov-03 | B/Cambodia/1164/2015 |
| EPI763107 | HA | Cambodia | 2015-Nov-05 | B/Cambodia/1179/2015 |
| EPI765341 | HA | Cambodia | 2015-Nov-18 | B/Cambodia/1224/2015 |
| EPI763115 | HA | Cambodia | 2015-Nov-17 | B/Cambodia/1226/2015 |
| EPI763152 | HA | Cambodia | 2015-Nov-23 | B/Cambodia/1250/2015 |
| EPI753797 | HA | Cambodia | 2015-Nov-25 | B/Cambodia/1257/2015 |
| EPI765317 | HA | Cambodia | 2015-Nov-30 | B/Cambodia/1273/2015 |
| EPI765349 | HA | Cambodia | 2015-Dec-02 | B/Cambodia/1275/2015 |
| EPI769745 | HA | Cambodia | 2015-Dec-03 | B/Cambodia/1284/2015 |
| EPI753805 | HA | Cambodia | 2015-Dec-02 | B/Cambodia/1287/2015 |
| EPI765333 | HA | Cambodia | 2015-Dec-07 | B/Cambodia/1297/2015 |
| EPI765325 | HA | Cambodia | 2015-Dec-08 | B/Cambodia/1302/2015 |
| EPI830971 | HA | Cambodia | 2015-Dec-13 | B/Cambodia/1314/2015 |
| EPI765357 | HA | Cambodia | 2015-Dec-21 | B/Cambodia/1334/2015 |
| EPI769753 | HA | Cambodia | 2015-Dec-23 | B/Cambodia/1355/2015 |
| EPI765180 | HA | Cambodia | 2015-Dec-30 | B/Cambodia/AD06593/2015 |
| EPI765182 | HA | Cambodia | 2015-Dec-02 | B/Cambodia/FSS29723/2015 |
| EPI765184 | HA | Cambodia | 2015-Oct-30 | B/Cambodia/Z1210534/2015 |
| EPI765166 | HA | Cambodia | 2012-Dec-04 | B/Cambodia/Z1212504/2015 |
| EPI765176 | HA | Cambodia | 2015-Dec-24 | B/Cambodia/Z1229504/2015 |
