# Supplementary material for: Circulation and characterization of seasonal influenza viruses in Cambodia, 2012‐2015
Source: Influenza Other Respir Viruses. 2019 Jun 28;13(5):465–76. doi: 10.1111/irv.12647 (PMC6692578; doi:10.1111/irv.12647)
Supplement: Supplementary file 2 [file IRV-13-465-s002.docx]

**Supplementary Table 2.** Age and gender distribution across the four testing years (2012-2015) for influenza-positive ILI patients

|  | **Influenza virus-infected ILI patients** | | |
| --- | --- | --- | --- |
| **Year** | **Average age, years**  **(minimal-maximal age)** | **Male (%)** | **Female (%)** |
| **2012** | 7.6 (3m-77y) | 57.9 | 42.1 |
| **2013** | 9.1 (1m-68y) | 59.4 | 40.6 |
| **2014** | 10.8 (11d-67y) | 57.9 | 42.1 |
| **2015** | 8.0 (11d-67y) | 50.6 | 49.4 |
| **4 years** | 8.8 (11d-77y) | 56.7 | 43.3 |
