# Supplementary material for: Circulation and characterization of seasonal influenza viruses in Cambodia, 2012‐2015
Source: Influenza Other Respir Viruses. 2019 Jun 28;13(5):465–76. doi: 10.1111/irv.12647 (PMC6692578; doi:10.1111/irv.12647)
Supplement: Supplementary file 3 [file IRV-13-465-s003.docx]

**Supplementary Table 3.** Cambodian influenza A/H3N2, A/H1N1pdm09 and influenza B virus Neuraminidase sequences included in the analyses are available via the GISAID website ([www.gisaid.org](http://www.gisaid.org))

| **GISAID Accession#** | | | **Segment** | **Country** | **Collection date** | **Isolate name** |
| --- | --- | --- | --- | --- | --- | --- |
| EPI394249 | | | NA | Cambodia | 2012-Jul-04 | A/Cambodia/5/2012 |
| EPI394252 | | | NA | Cambodia | 2012-Jul-19 | A/Cambodia/10/2012 |
| EPI394321 | | | NA | Cambodia | 2012-Jun-27 | A/Cambodia/31/2012 |
| EPI743830 | | | NA | Cambodia | 2012-Aug-28 | A/Cambodia/W0908340/2012 |
| EPI743835 | | | NA | Cambodia | 2012-Sep-10 | A/Cambodia/W0921311/2012 |
| EPI743840 | | | NA | Cambodia | 2012-Oct-05 | A/Cambodia/W1023343/2012 |
| EPI743845 | | | NA | Cambodia | 2012-Oct-10 | A/Cambodia/W1023347/2012 |
| EPI743850 | | | NA | Cambodia | 2012-Oct-18 | A/Cambodia/W1023353/2012 |
| EPI743855 | | | NA | Cambodia | 2012-Oct-18 | A/Cambodia/W1023355/2012 |
| EPI491282 | | | NA | Cambodia | 2013-Jul-09 | A/Cambodia/X0717310/2013 |
| EPI541599 | | | NA | Cambodia | 2014-Jun-02 | A/Cambodia/653/2014 |
| EPI551517 | | | NA | Cambodia | 2014-Jun-16 | A/Cambodia/FSS28237/2014 |
| EPI551523 | | | NA | Cambodia | 2014-Jun-19 | A/Cambodia/Y0703302/2014 |
| EPI551528 | | | NA | Cambodia | 2014-May-28 | A/Cambodia/Y0721397/2014 |
| EPI551532 | | | NA | Cambodia | 2014-Jun-26 | A/Cambodia/Y0721447/2014 |
| EPI575157 | | | NA | Cambodia | 2014-Dec-17 | A/Cambodia/1384/2014 |
| EPI575343 | | | NA | Cambodia | 2014-Dec-11 | A/Cambodia/1370/2014 |
| EPI575351 | | | NA | Cambodia | 2014-Dec-11 | A/Cambodia/1372/2014 |
| EPI578857 | | | NA | Cambodia | 2014-Aug-05 | A/Cambodia/870/2014 |
| EPI579365 | | | NA | Cambodia | 2014-Nov-11 | A/Cambodia/1244/2014 |
| EPI612261 | | | NA | Cambodia | 2014-Dec-13 | A/Cambodia/1393/2014 |
| EPI629749 | | | NA | Cambodia | 2014-Oct-18 | A/Cambodia/AD04410/2014 |
| EPI629752 | | | NA | Cambodia | 2014-Nov-12 | A/Cambodia/AD04526/2014 |
| EPI629755 |  |  | NA | Cambodia 2014-Sep-30 A/Cambodia/FSS28296/2014 | | |
| EPI629758 | | | NA | Cambodia | 2014-Nov-26 | A/Cambodia/Y1204310/2014 |
| EPI629761 | | | NA | Cambodia | 2014-Dec-02 | A/Cambodia/Y1218307/2014 |
| EPI648838 | | | NA | Cambodia | 2015-Jun-29 | A/Cambodia/Z0709311/2015 |
| EPI648841 | | | NA | Cambodia | 2015-Jun-08 | A/Cambodia/Z0727331/2015 |
| EPI652570 | | | NA | Cambodia | 2015-Jun-11 | A/Cambodia/Z0727320/2015 |
| EPI652573 | | | NA | Cambodia | 2015-Jun-25 | A/Cambodia/Z0727326/2015 |
| EPI652576 | | | NA | Cambodia | 2015-Jun-29 | A/Cambodia/Z0727330/2015 |
| EPI652579 | | | NA | Cambodia | 2015-Jul-15 | A/Cambodia/Z0722378/2015 |
| EPI652582 | | | NA | Cambodia | 2015-Jun-28 | A/Cambodia/Z0727325/2015 |
| EPI652591 | | | NA | Cambodia | 2015-Jun-24 | A/Cambodia/Ad05323/2015 |
| EPI652594 | | | NA | Cambodia | 2015-Jul-02 | A/Cambodia/Z0709313/2015 |
| EPI652597 | | | NA | Cambodia | 2015-Jul-14 | A/Cambodia/Z0722377/2015 |
| EPI652600 | | | NA | Cambodia | 2015-Jul-15 | A/Cambodia/Z0722379/2015 |
| EPI652603 | | | NA | Cambodia | 2015-Jul-14 | A/Cambodia/Z0722380/2015 |
| EPI652606 | | | NA | Cambodia | 2015-Jun-29 | A/Cambodia/Z0727328/2015 |
| EPI652609 | | | NA | Cambodia | 2015-Jun-29 | A/Cambodia/Z0727329/2015 |
| EPI652612 | | | NA | Cambodia | 2015-Jul-01 | A/Cambodia/Z0709310/2015 |
| EPI652615 | | | NA | Cambodia | 2015-Jul-01 | A/Cambodia/Z0722381/2015 |
| EPI676115 | | | NA | Cambodia | 2015-May-26 | A/Cambodia/Z0727327/2015 |
| EPI676118 | | | NA | Cambodia | 2015-Jun-24 | A/Cambodia/Z0727323/2015 |
| EPI676121 | | | NA | Cambodia | 2015-Jun-29 | A/Cambodia/Z0709312/2015 |
| EPI676124 | | | NA | Cambodia | 2015-Jun-18 | A/Cambodia/Ad05096/2015 |
| EPI702039 | | | NA | Cambodia | 2015-Aug-31 | A/Cambodia/0924/2015 |
| EPI702134 | | | NA | Cambodia | 2015-Aug-27 | A/Cambodia/0911/2015 |
| EPI711010 | | | NA | Cambodia | 2015-Aug-26 | A/Cambodia/0909/2015 |
| EPI711018 | | | NA | Cambodia | 2015-Sep-01 | A/Cambodia/0929/2015 |
| EPI711026 | | | NA | Cambodia | 2015-Oct-28 | A/Cambodia/1137/2015 |
| EPI711281 | | | NA | Cambodia | 2015-Aug-05 | A/Cambodia/0840/2015 |
| EPI711289 | | | NA | Cambodia | 2015-Aug-07 | A/Cambodia/0842/2015 |
| EPI711297 | | | NA | Cambodia | 2015-Aug-13 | A/Cambodia/0861/2015 |
| EPI711305 | | | NA | Cambodia | 2015-Aug-25 | A/Cambodia/0895/2015 |
| EPI711313 | | | NA | Cambodia | 2015-Aug-26 | A/Cambodia/0917/2015 |
| EPI711321 | | | NA | Cambodia | 2015-Sep-02 | A/Cambodia/0942/2015 |
| EPI711329 | | | NA | Cambodia | 2015-Sep-08 | A/Cambodia/0951/2015 |
| EPI712417 | | | NA | Cambodia | 2015-Aug-14 | A/Cambodia/0869/2015 |
| EPI712425 | | | NA | Cambodia | 2015-Aug-24 | A/Cambodia/0887/2015 |
| EPI712433 | | | NA | Cambodia | 2015-Aug-26 | A/Cambodia/0909/2015 |
| EPI715252 | | | NA | Cambodia | 2015-Aug-18 | A/Cambodia/0877/2015 |
| EPI727323 | | | NA | Cambodia | 2015-Nov-11 | A/Cambodia/1201/2015 |
| EPI727335 | | | NA | Cambodia | 2015-Dec-03 | A/Cambodia/1290/2015 |
| EPI730017 | | | NA | Cambodia | 2015-Nov-11 | A/Cambodia/1181/2015 |
| EPI746658 | | | NA | Cambodia | 2015-Sep-22 | A/Cambodia/0009/2015 |
| EPI765130 | | | NA | Cambodia | 2015-Dec-08 | A/Cambodia/FSS31758/2015 |
| EPI868900 | | | NA | Cambodia | 2015-Nov-03 | A/Cambodia/FSS31603/2015 |
| EPI443610 | | | NA | Cambodia | 2013-Jan-04 | A/Cambodia/13/2013 |
| EPI443613 | | | NA | Cambodia | 2013-Jan-23 | A/Cambodia/10077/2013 |
| EPI443616 | | | NA | Cambodia | 2013-Jan-04 | A/Cambodia/13/2013 |
| EPI491258 | | | NA | Cambodia | 2013-Jan-22 | A/Cambodia/X0206305/2013 |
| EPI491261 | | | NA | Cambodia | 2013-May-22 | A/Cambodia/X0522305/2013 |
| EPI491288 | | | NA | Cambodia | 2013-Jun-26 | A/Cambodia/X0717301/2013 |
| EPI491291 | | | NA | Cambodia | 2013-Jul-12 | A/Cambodia/X0717333/2013 |
| EPI491541 | | | NA | Cambodia | 2013-Jul-03 | A/Cambodia/X0717312/2013 |
| EPI529446 | | | NA | Cambodia | 2013-Sep-13 | A/Cambodia/X0930306/2013 |
| EPI529449 | | | NA | Cambodia | 2013-Oct-09 | A/Cambodia/X1104315/2013 |
| EPI541526 | | | NA | Cambodia | 2014-Jun-04 | A/Cambodia/0671/2014 |
| EPI541529 | | | NA | Cambodia | 2014-Jun-26 | A/Cambodia/0745/2014 |
| EPI541532 | | | NA | Cambodia | 2014-Jun-26 | A/Cambodia/0737/2014 |
| EPI551355 | | | NA | Cambodia | 2014-Jun-11 | A/Cambodia/Y0630302/2014 |
| EPI551358 | | | NA | Cambodia | 2014-Jun-26 | A/Cambodia/Y0721444/2014 |
| EPI565258 | | | NA | Cambodia | 2014-Dec-05 | A/Cambodia/Y1218309/2014 |
| EPI636077 | | | NA | Cambodia | 2015-Jun-16 | A/Cambodia/FSS39385/2015 |
| EPI697809 | | | NA | Cambodia | 2015-Sep-12 | A/Cambodia/0981/2015 |
| EPI697817 | | | NA | Cambodia | 2015-Sep-25 | A/Cambodia/1021/2015 |
| EPI759327 | | | NA | Cambodia | 2015-Nov-05 | A/Cambodia/1191/2015 |
| EPI759335 | | | NA | Cambodia | 2015-Dec-09 | A/Cambodia/1300/2015 |
| EPI759343 | | | NA | Cambodia | 2015-Dec-15 | A/Cambodia/1326/2015 |
| EPI759351 | | | NA | Cambodia | 2015-Dec-21 | A/Cambodia/1340/2015 |
| EPI762478 | | | NA | Cambodia | 2015-Nov-05 | A/Cambodia/1191/2015 |
| EPI765116 | | | NA | Cambodia | 2015-Sep-25 | A/Cambodia/Z1210510/2015 |
| EPI417347 | | | NA | Cambodia | 2012-Apr-11 | B/Cambodia/2/2012 |
| EPI491276 | | | NA | Cambodia | 2013-May-29 | B/Cambodia/X0611302/2013 |
| EPI529382 | | | NA | Cambodia | 2013-Nov-13 | B/Cambodia/X1126361/2013 |
| EPI529385 | | | NA | Cambodia | 2013-Nov-25 | B/Cambodia/X1126384/2013 |
| EPI540559 | | | NA | Cambodia | 2014-May-03 | B/Cambodia/538/2014 |
| EPI551251 | | | NA | Cambodia | 2014-May-19 | B/Cambodia/Y0721371/2014 |
| EPI562020 | | | NA | Cambodia | 2014-Nov-11 | B/Cambodia/Fss26970/2014 |
| EPI562023 | | | NA | Cambodia | 2014-Dec-08 | B/Cambodia/FSS29374/2014 |
| EPI562026 | | | NA | Cambodia | 2014-Aug-09 | B/Cambodia/Y0827324/2014 |
| EPI582431 | | | NA | Cambodia | 2014-Nov-17 | B/Cambodia/1269/2014 |
| EPI582435 | | | NA | Cambodia | 2014-Nov-13 | B/Cambodia/1253/2014 |
| EPI630027 | | | NA | Cambodia | 2014-Nov-02 | B/Cambodia/AD04506/2014 |
| EPI696756 | | | NA | Cambodia | 2015-Aug-21 | B/Cambodia/0894/2015 |
| EPI696764 | | | NA | Cambodia | 2015-Sep-23 | B/Cambodia/1011/2015 |
| EPI696772 | | | NA | Cambodia | 2015-Oct-07 | B/Cambodia/1072/2015 |
| EPI696780 | | | NA | Cambodia | 2015-Oct-06 | B/Cambodia/1087/2015 |
| EPI696788 | | | NA | Cambodia | 2015-Oct-21 | B/Cambodia/1122/2015 |
| EPI696796 | | | NA | Cambodia | 2015-Oct-22 | B/Cambodia/1123/2015 |
| EPI696804 | | | NA | Cambodia | 2015-Oct-27 | B/Cambodia/1141/2015 |
| EPI753796 | | | NA | Cambodia | 2015-Nov-25 | B/Cambodia/1257/2015 |
| EPI753804 | | | NA | Cambodia | 2015-Dec-02 | B/Cambodia/1287/2015 |
| EPI753811 | | | NA | Cambodia | 2015-Dec-30 | B/Cambodia/0004/2015 |
| EPI753818 | | | NA | Cambodia | 2015-Nov-30 | B/Cambodia/1273/2015 |
| EPI763098 | | | NA | Cambodia | 2015-Dec-08 | B/Cambodia/1302/2015 |
| EPI763106 | | | NA | Cambodia | 2015-Nov-05 | B/Cambodia/1179/2015 |
| EPI763114 | | | NA | Cambodia | 2015-Nov-17 | B/Cambodia/1226/2015 |
| EPI763121 | | | NA | Cambodia | 2015-Dec-07 | B/Cambodia/1297/2015 |
| EPI763128 | | | NA | Cambodia | 2015-Nov-18 | B/Cambodia/1224/2015 |
| EPI763135 | | | NA | Cambodia | 2015-Dec-02 | B/Cambodia/1275/2015 |
| EPI763143 | | | NA | Cambodia | 2015-Nov-03 | B/Cambodia/1164/2015 |
| EPI763151 | | | NA | Cambodia | 2015-Nov-23 | B/Cambodia/1250/2015 |
| EPI763158 | | | NA | Cambodia | 2015-Dec-21 | B/Cambodia/1334/2015 |
| EPI765165 | | | NA | Cambodia | 2012-Dec-04 | B/Cambodia/Z1212504/2015 |
| EPI765175 | | | NA | Cambodia | 2015-Dec-24 | B/Cambodia/Z1229504/2015 |
| EPI765179 | | | NA | Cambodia | 2015-Dec-30 | B/Cambodia/AD06593/2015 |
| EPI765181 | | | NA | Cambodia | 2015-Dec-02 | B/Cambodia/FSS29723/2015 |
| EPI765183 | | | NA | Cambodia | 2015-Oct-30 | B/Cambodia/Z1210534/2015 |
| EPI765308 | | | NA | Cambodia | 2015-Dec-30 | B/Cambodia/0004/2015 |
| EPI765316 | | | NA | Cambodia | 2015-Nov-30 | B/Cambodia/1273/2015 |
| EPI765324 | | | NA | Cambodia | 2015-Dec-08 | B/Cambodia/1302/2015 |
| EPI765332 | | | NA | Cambodia | 2015-Dec-07 | B/Cambodia/1297/2015 |
| EPI765340 | | | NA | Cambodia | 2015-Nov-18 | B/Cambodia/1224/2015 |
| EPI765348 | | | NA | Cambodia | 2015-Dec-02 | B/Cambodia/1275/2015 |
| EPI765356 | | | NA | Cambodia | 2015-Dec-21 | B/Cambodia/1334/2015 |
| EPI769744 | | | NA | Cambodia | 2015-Dec-03 | B/Cambodia/1284/2015 |
| EPI769752 | | | NA | Cambodia | 2015-Dec-23 | B/Cambodia/1355/2015 |
| EPI830970 | | | NA | Cambodia | 2015-Dec-13 | B/Cambodia/1314/2015 |
| EPI869135 | | | NA | Cambodia | 2015-Nov-30 | B/Cambodia/Z1212502/2015 |
