# Supplementary material for: Circulation and characterization of seasonal influenza viruses in Cambodia, 2012‐2015
Source: Influenza Other Respir Viruses. 2019 Jun 28;13(5):465–76. doi: 10.1111/irv.12647 (PMC6692578; doi:10.1111/irv.12647)
Supplement: Supplementary file 4 [file IRV-13-465-s004.docx]

**Supplementary Table 4.** Antiviral resistance results from Cambodian seasonal influenza isolates, 2012-2015

|  | **Adamantanes** | | **Oseltamivir** | | **Zanamivir** | |
| --- | --- | --- | --- | --- | --- | --- |
| **Virus type and subtype** | **Isolates tested^a^** | **Resistant**^a^ | **Isolates tested** | **Resistant** | **Isolates tested** | **Resistant** |
| A/H3N2 | 66 | 66 | 148 | 0 | 148 | 0 |
| A/H1N1pdm09 | 27 | 27 | 73 | 0 | 73 | 0 |
| Influenza B | N/A | N/A | 83 | 0 | 83 | 0 |

^a^ resistance to adamantanes was inferred by sequence analysis of the M2 gene. Isolates with asparagine at position 31 were predicted to be resistant to adamantanes
