# Supplementary material for: Circulation and characterization of seasonal influenza viruses in Cambodia, 2012‐2015
Source: Influenza Other Respir Viruses. 2019 Jun 28;13(5):465–76. doi: 10.1111/irv.12647 (PMC6692578; doi:10.1111/irv.12647)
Supplement: Supplementary file 5 [file IRV-13-465-s005.docx]

**Supplementary Table 5.** Cambodian influenza A/H3N2, A/H1N1pdm09 and influenza B virus Matrix sequences included in the analyses are available via the GISAID website ([www.gisaid.org](http://www.gisaid.org))

| **GISAID Accession#** | **Segment** | **Country** | **Collection date** | **Isolate name** |
| --- | --- | --- | --- | --- |
| EPI394248 | M | Cambodia | 2012-Jul-04 | A/Cambodia/5/2012 |
| EPI394251 | M | Cambodia | 2012-Jul-19 | A/Cambodia/10/2012 |
| EPI394320 | M | Cambodia | 2012-Jun-27 | A/Cambodia/31/2012 |
| EPI743831 | M | Cambodia | 2012-Aug-28 | A/Cambodia/W0908340/2012 |
| EPI743836 | M | Cambodia | 2012-Sep-12 | A/Cambodia/W0921311/2012 |
| EPI743841 | M | Cambodia | 2012-Oct-05 | A/Cambodia/W1023343/2012 |
| EPI743846 | M | Cambodia | 2012-Oct-10 | A/Cambodia/W1023347/2012 |
| EPI743851 | M | Cambodia | 2012-Oct-18 | A/Cambodia/W1023353/2012 |
| EPI743856 | M | Cambodia | 2012-Oct-18 | A/Cambodia/W1023355/2012 |
| EPI491281 | M | Cambodia | 2013-Jul-09 | A/Cambodia/X0717310/2013 |
| EPI541598 | M | Cambodia | 2014-Jun-02 | A/Cambodia/653/2014 |
| EPI578853 | M | Cambodia | 2014-Aug-05 | A/Cambodia/870/2014 |
| EPI579362 | M | Cambodia | 2014-Nov-11 | A/Cambodia/1244/2014 |
| EPI575339 | M | Cambodia | 2014-Dec-11 | A/Cambodia/1370/2014 |
| EPI575347 | M | Cambodia | 2014-Dec-11 | A/Cambodia/1372/2014 |
| EPI575153 | M | Cambodia | 2014-Dec-17 | A/Cambodia/1384/2014 |
| EPI612259 | M | Cambodia | 2014-Dec-13 | A/Cambodia/1393/2014 |
| EPI629748 | M | Cambodia | 2014-Oct-18 | A/Cambodia/AD04410/2014 |
| EPI629751 | M | Cambodia | 2014-Nov-12 | A/Cambodia/AD04526/2014 |
| EPI551514 | M | Cambodia | 2014-Jun-16 | A/Cambodia/FSS28237/2014 |
| EPI629754 | M | Cambodia | 2014-Sep-30 | A/Cambodia/FSS28296/2014 |
| EPI551521 | M | Cambodia | 2014-Jun-19 | A/Cambodia/Y0703302/2014 |
| EPI551527 | M | Cambodia | 2014-May-28 | A/Cambodia/Y0721397/2014 |
| EPI551531 | M | Cambodia | 2014-Jun-26 | A/Cambodia/Y0721447/2014 |
| EPI629757 | M | Cambodia | 2014-Nov-26 | A/Cambodia/Y1204310/2014 |
| EPI629760 | M | Cambodia | 2014-Dec-02 | A/Cambodia/Y1218307/2014 |
| EPI746654 | M | Cambodia | 2015-Sep-22 | A/Cambodia/0009/2015 |
| EPI711277 | M | Cambodia | 2015-Aug-05 | A/Cambodia/0840/2015 |
| EPI711285 | M | Cambodia | 2015-Aug-07 | A/Cambodia/0842/2015 |
| EPI711293 | M | Cambodia | 2015-Aug-13 | A/Cambodia/0861/2015 |
| EPI712413 | M | Cambodia | 2015-Aug-14 | A/Cambodia/0869/2015 |
| EPI715248 | M | Cambodia | 2015-Aug-18 | A/Cambodia/0877/2015 |
| EPI712421 | M | Cambodia | 2015-Aug-24 | A/Cambodia/0887/2015 |
| EPI711301 | M | Cambodia | 2015-Aug-25 | A/Cambodia/0895/2015 |
| EPI711006 | M | Cambodia | 2015-Aug-26 | A/Cambodia/0909/2015 |
| EPI702130 | M | Cambodia | 2015-Aug-27 | A/Cambodia/0911/2015 |
| EPI711309 | M | Cambodia | 2015-Aug-26 | A/Cambodia/0917/2015 |
| EPI702035 | M | Cambodia | 2015-Aug-31 | A/Cambodia/0924/2015 |
| EPI711014 | M | Cambodia | 2015-Sep-01 | A/Cambodia/0929/2015 |
| EPI711014 | M | Cambodia | 2015-Sep-02 | A/Cambodia/0942/2015 |
| EPI711325 | M | Cambodia | 2015-Sep-08 | A/Cambodia/0951/2015 |
| EPI711022 | M | Cambodia | 2015-Oct-28 | A/Cambodia/1137/2015 |
| EPI730013 | M | Cambodia | 2015-Nov-11 | A/Cambodia/1181/2015 |
| EPI727318 | M | Cambodia | 2015-Nov-11 | A/Cambodia/1201/2015 |
| EPI727329 | M | Cambodia | 2015-Dec-03 | A/Cambodia/1290/2015 |
| EPI676123 | M | Cambodia | 2015-Jun-18 | A/Cambodia/Ad05096/2015 |
| EPI652590 | M | Cambodia | 2015-Jun-24 | A/Cambodia/Ad05323/2015 |
| EPI765129 | M | Cambodia | 2015-Dec-08 | A/Cambodia/FSS31758/2015 |
| EPI652611 | M | Cambodia | 2015-Jul-01 | A/Cambodia/Z0709310/2015 |
| EPI648837 | M | Cambodia | 2015-Jun-26 | A/Cambodia/Z0709311/2015 |
| EPI676120 | M | Cambodia | 2015-Jun-29 | A/Cambodia/Z0709312/2015 |
| EPI652593 | M | Cambodia | 2015-Jul-02 | A/Cambodia/Z0709313/2015 |
| EPI652596 | M | Cambodia | 2015-Jul-14 | A/Cambodia/Z0722377/2015 |
| EPI652578 | M | Cambodia | 2015-Jul-15 | A/Cambodia/Z0722378/2015 |
| EPI652599 | M | Cambodia | 2015-Jul-15 | A/Cambodia/Z0722379/2015 |
| EPI652602 | M | Cambodia | 2015-Jul-14 | A/Cambodia/Z0722380/2015 |
| EPI652614 | M | Cambodia | 2015-Jul-01 | A/Cambodia/Z0722381/2015 |
| EPI652569 | M | Cambodia | 2015-Jun-11 | A/Cambodia/Z0727320/2015 |
| EPI676117 | M | Cambodia | 2015-Jun-24 | A/Cambodia/Z0727323/2015 |
| EPI652581 | M | Cambodia | 2015-Jun-28 | A/Cambodia/Z0727325/2015 |
| EPI652572 | M | Cambodia | 2015-Jun-25 | A/Cambodia/Z0727326/2015 |
| EPI676114 | M | Cambodia | 2015-May-26 | A/Cambodia/Z0727327/2015 |
| EPI652605 | M | Cambodia | 2015-Jun-29 | A/Cambodia/Z0727328/2015 |
| EPI652608 | M | Cambodia | 2015-Jun-29 | A/Cambodia/Z0727329/2015 |
| EPI652575 | M | Cambodia | 2015-Jun-29 | A/Cambodia/Z0727330/2015 |
| EPI648840 | M | Cambodia | 2015-Jun-08 | A/Cambodia/Z0727331/2015 |
| EPI743948 | M | Cambodia | 2012-Jul-18 | A/Cambodia/W0908339/2012 |
| EPI743953 | M | Cambodia | 2012-Oct-08 | A/Cambodia/W1023346/2012 |
| EPI743968 | M | Cambodia | 2012-Oct-11 | A/Cambodia/W1023349/2012 |
| EPI743971 | M | Cambodia | 2012-Oct-19 | A/Cambodia/W1023356/2012 |
| EPI447186 | M | Cambodia | 2013-Jan-04 | A/Cambodia/13/2013 |
| EPI443612 | M | Cambodia | 2013-Jan-23 | A/Cambodia/10077/2013 |
| EPI491257 | M | Cambodia | 2013-Jan-22 | A/Cambodia/X0206305/2013 |
| EPI491260 | M | Cambodia | 2013-May-22 | A/Cambodia/X0522305/2013 |
| EPI491287 | M | Cambodia | 2013-Jun-22 | A/Cambodia/X0717301/2013 |
| EPI491540 | M | Cambodia | 2013-Jul-03 | A/Cambodia/X0717312/2013 |
| EPI491290 | M | Cambodia | 2013-Jul-12 | A/Cambodia/X0717333/2013 |
| EPI529445 | M | Cambodia | 2013-Sep-13 | A/Cambodia/X0930306/2013 |
| EPI529448 | M | Cambodia | 2013-Oct-09 | A/Cambodia/X1104315/2013 |
| EPI541525 | M | Cambodia | 2014-Jun-04 | A/Cambodia/0671/2014 |
| EPI541531 | M | Cambodia | 2014-Jun-26 | A/Cambodia/0737/2014 |
| EPI541528 | M | Cambodia | 2014-Jun-26 | A/Cambodia/0745/2014 |
| EPI551354 | M | Cambodia | 2014-Jun-11 | A/Cambodia/Y0630302/2014 |
| EPI551357 | M | Cambodia | 2014-Jun-26 | A/Cambodia/Y0721444/2014 |
| EPI565257 | M | Cambodia | 2014-Dec-05 | A/Cambodia/Y1218309/2014 |
| EPI697805 | M | Cambodia | 2015-Sep-12 | A/Cambodia/0981/2015 |
| EPI697813 | M | Cambodia | 2015-Sep-25 | A/Cambodia/1021/2015 |
| EPI759323 | M | Cambodia | 2015-Nov-05 | A/Cambodia/1191/2015 |
| EPI759331 | M | Cambodia | 2015-Dec-09 | A/Cambodia/1300/2015 |
| EPI759339 | M | Cambodia | 2015-Dec-15 | A/Cambodia/1326/2015 |
| EPI759347 | M | Cambodia | 2015-Dec-21 | A/Cambodia/1340/2015 |
| EPI636076 | M | Cambodia | 2015-Jun-16 | A/Cambodia/FSS39385/2015 |
| EPI765115 | M | Cambodia | 2015-Sep-25 | A/Cambodia/Z1210510/2015 |
| EPI450454 | M | Cambodia | 2012-Apr-11 | B/Cambodia/2/2012 |
| EPI582434 | M | Cambodia | 2014-Nov-13 | B/Cambodia/1253/2014 |
| EPI582428 | M | Cambodia | 2014-Nov-17 | B/Cambodia/1269/2014 |
| EPI638853 | M | Cambodia | 2014-Dec-08 | B/Cambodia/FSS29374/2014 |
| EPI765304 | M | Cambodia | 2015-Dec-30 | B/Cambodia/0004/2015 |
| EPI696752 | M | Cambodia | 2015-Aug-21 | B/Cambodia/0894/2015 |
| EPI696760 | M | Cambodia | 2015-Sep-23 | B/Cambodia/1011/2015 |
| EPI696768 | M | Cambodia | 2015-Oct-07 | B/Cambodia/1072/2015 |
| EPI696776 | M | Cambodia | 2015-Oct-06 | B/Cambodia/1087/2015 |
| EPI696784 | M | Cambodia | 2015-Oct-21 | B/Cambodia/1122/2015 |
| EPI696792 | M | Cambodia | 2015-Oct-22 | B/Cambodia/1123/2015 |
| EPI696800 | M | Cambodia | 2015-Oct-27 | B/Cambodia/1141/2015 |
| EPI763139 | M | Cambodia | 2015-Nov-03 | B/Cambodia/1164/2015 |
| EPI763102 | M | Cambodia | 2015-Nov-05 | B/Cambodia/1179/2015 |
| EPI765336 | M | Cambodia | 2015-Nov-18 | B/Cambodia/1224/2015 |
| EPI763110 | M | Cambodia | 2015-Nov-17 | B/Cambodia/1226/2015 |
| EPI763147 | M | Cambodia | 2015-Nov-23 | B/Cambodia/1250/2015 |
| EPI753792 | M | Cambodia | 2015-Nov-25 | B/Cambodia/1257/2015 |
| EPI765312 | M | Cambodia | 2015-Nov-30 | B/Cambodia/1273/2015 |
| EPI765344 | M | Cambodia | 2015-Dec-02 | B/Cambodia/1275/2015 |
| EPI769740 | M | Cambodia | 2015-Dec-03 | B/Cambodia/1284/2015 |
| EPI753800 | M | Cambodia | 2015-Dec-02 | B/Cambodia/1287/2015 |
| EPI765328 | M | Cambodia | 2015-Dec-07 | B/Cambodia/1297/2015 |
| EPI765320 | M | Cambodia | 2015-Dec-08 | B/Cambodia/1302/2015 |
| EPI830966 | M | Cambodia | 2015-Dec-13 | B/Cambodia/1314/2015 |
| EPI765352 | M | Cambodia | 2015-Dec-21 | B/Cambodia/1334/2015 |
| EPI769748 | M | Cambodia | 2015-Dec-23 | B/Cambodia/1355/2015 |
| EPI816378 | M | Cambodia | 2015-Dec-02 | B/Cambodia/FSS29723/2015 |
| EPI816343 | M | Cambodia | 2012-Dec-04 | B/Cambodia/Z1212504/2015 |
